# Cyclodextrin-Assisted Extraction and Encapsulation of Parthenolide- and Polyphenol-Rich Feverfew (*Tanacetum parthenium* L.) for Enhanced Bioactivity

**DOI:** 10.3390/ph19081176

**Published:** 2026-07-27

**Authors:** Nada Ćujić Nikolić, Dajana Lazarević, Aleksandra Jovanović, Jelena Jovanović, Katarina Šavikin, Nibina Nizar, Gorana Ilić, Goran Vladisavljević, Tatjana Trtić-Petrović

**Affiliations:** 1 Institute for Medicinal Plants Research ‘’Dr. Josif Pančić’’, 11000 Belgrade, Serbia; ncujic@mocbilja.rs (N.Ć.N.);; 2 Vinča Institute of Nuclear Sciences, National Institute of the Republic of Serbia, University of Belgrade, 11000 Belgrade, Serbiattrtic@vinca.rs (T.T.-P.); 3 Institute for the Application of Nuclear Energy INEP, University of Belgrade, 11080 Belgrade, Serbia; ajovanovic@inep.co.rs (A.J.); gorana.ilic@inep.co.rs (G.I.); 4 Chemical Engineering Department, Loughborough University, Loughborough LE11 3TU, UKg.vladisavljevic@lboro.ac.uk (G.V.)

**Keywords:** feverfew (*Tanacetum parthenium* L.), cyclodextrin, extraction, encapsulation, spray drying, antioxidant, anti-inflammatory

## Abstract

**Background/Objectives**: The extraction and encapsulation of *Tanacetum parthenium* L. (feverfew) represent highly relevant approaches for maximizing the recovery and protection of its bioactive phytocomplexes, particularly the main active compound, parthenolide (PAR). The present study aimed to develop and optimize a cyclodextrin-based extraction and encapsulation method for *T. parthenium* to improve the chemical and physicochemical properties and biological activity of bioactive-rich phytocomplexes. **Methods**: The extraction process was optimized by comparing conventional maceration (ME) and ultrasound-assisted extraction (UE), using different ethanol–water mixtures as extractants, with and without hydroxypropyl-β-cyclodextrin (CD) as an extraction enhancer. The highest PAR yield (approximately 6.3 mg/g dried biomass), combined with enhanced total polyphenol and flavonoid contents, was achieved using 50% (*v*/*v*) ethanol with 0.5% CD under UE. The optimized extract was spray-dried to produce powder formulations, with or without carrier material, which have been characterized for their technological, chemical, physicochemical, structural, and biological properties. **Results**: The inclusion of CD significantly improved process yield (approximately 65% compared to 47% for the non-encapsulated extract) and reduced residual moisture content, indicating enhanced drying efficiency and improved powder stability. Furthermore, the encapsulated formulation demonstrated preserved antioxidant and anti-inflammatory activity in cell-based assays. **Conclusions**: The developed CD-based encapsulation method represents a promising strategy for stabilizing feverfew bioactives and enhancing their potential applicability in pharmaceutical and dermo-cosmetic formulations.

## 1. Introduction

*Tanacetum parthenium* L. (feverfew) is a perennial herb in the family Asteraceae, traditionally used in medicine [1,2]. Native to southeastern Europe and the Balkan Peninsula, it is now widely naturalized and cultivated across Europe, North America, and parts of Asia [3]. Its dried leaves and extracts are frequently used in traditional and modern herbal medicine, particularly for fever, migraine, rheumatic diseases, and various inflammatory disorders [1,2]. In some countries, feverfew supplements rank among the most commonly used over-the-counter herbal remedies for headache prevention [4,5]. Its therapeutic potential is primarily attributed to a diverse profile of bioactive compounds, including sesquiterpene lactones (notably parthenolide—PAR), flavonoids, and volatile oils [4,6]. Among these bioactives, PAR stands out as the most pharmacologically significant constituent, exhibiting potent anti-inflammatory, anti-migraine, and antitumor activities [7]. Although PAR exhibits considerable pharmacological relevance, its chemical instability and high sensitivity to light, heat, and oxygen [8], together with low aqueous solubility and poor oral bioavailability, limit its therapeutic application in conventional delivery systems [9]. All the above-mentioned limitations are also associated with polyphenolic compounds, which are present at high concentrations in feverfew. These physicochemical drawbacks necessitate the development of strategies and innovative formulations to enhance the preservation and biological potential of PAR and other bioactives.

One promising approach to overcoming these limitations is microencapsulation, a technique that can improve the stability of plant-derived bioactives and enhance the characteristics of liquid extract [10]. Among the various encapsulation techniques, spray drying has emerged as a particularly attractive method due to its cost-effectiveness, scalability, and compatibility with a wide range of bioactive compounds and carriers [11]. This technique enables the production of fine dried powders with improved handling properties, while focusing on preserving bioactives and enhancing chemical and physicochemical characteristics [12]. Nonetheless, the high inlet temperatures used during spray drying may pose a risk of thermal degradation, particularly for heat-sensitive compounds such as PAR, and may also result in variable encapsulation efficiency [13]. Potential process limitations include reduced yield due to material deposition on the drying chamber walls, solvent compatibility constraints, and technical equipment complexity, which may occasionally lead to operational challenges. However, these limitations can be effectively addressed through the careful selection of an appropriate carrier material, enabling improved process performance [14]. The choice of encapsulating agent plays a pivotal role in the success of spray drying, particularly the use of lower inlet temperatures [15]. In this study, hydroxypropyl-β-cyclodextrin (HPβCD, in further text referred to as CD) was selected as both an extraction agent (co-solvent) to enhance extraction efficiency and a carrier material due to its excellent ability to form an inclusion complex with lipophilic molecules, such as PAR and other bioactive compounds. This carrier is a water-soluble derivative of β-cyclodextrin with enhanced aqueous solubility, biocompatibility, and low toxicity [16]. CD improves the solubility, stability, and permeability of poorly soluble compounds, making it an adequate excipient for various formulations [17]. Complexation with CD may decrease the bioactive compound’s affinity for taste receptors, thereby improving palatability and patient acceptability [18], which is important for the bitter taste of feverfew extract. Although CD has previously been investigated both as an extraction-enhancing agent for several plant species [19,20,21] and as a carrier for the encapsulation of plant-derived bioactives [10,17,22], no previous study has systematically combined these two approaches into a single, coherent technological strategy. Herein, CD was employed to form inclusion complexes with bioactives from *T. parthenium*, with the aim of enhancing both the extraction efficiency and encapsulation performance. This dual approach was designed to improve feverfew’s biological activities and the functional properties of the obtained formulation. Our previous study [10] investigated the encapsulation of *Helichrysum plicatum* extract using CDs via spray drying and demonstrated the potential of such systems to enhance the pharmacological value of plant extracts.

This study aimed to develop encapsulated powder formulations of *Tanacetum parthenium* L. extract by spray drying using cyclodextrins (CDs), following an extraction strategy employing the same biopolymer system. The work focuses on the efficient extraction and stabilization of bioactive constituents, with parthenolide (PAR) as a representative marker compound, in order to enhance their chemical stability, physicochemical properties, and overall potential for therapeutic application. To the best of our knowledge and based on the available literature, the combined use of cyclodextrins as both an extraction co-solvent and encapsulating agent for *T. parthenium* L. has not been previously reported. Therefore, the principal novelty of this work lies in the development of a unified extraction–encapsulation platform in which hydroxypropyl-β-cyclodextrin simultaneously enhances phytochemical extraction and functions as the encapsulating matrix.

The resulting encapsulates were evaluated in terms of their physicochemical characteristics, and chemical profiling was performed using HPLC and UV–Vis spectrophotometry. In addition, a comprehensive assessment of biological activity, including antioxidant, anti-inflammatory, and cytotoxic assays, was conducted to establish correlations between phytochemical composition and pharmacological effects. By integrating extraction optimization, formulation development, and biological validation, this study contributes to the rational design of cyclodextrin-based delivery systems with potential pharmaceutical and cosmetic applications.

## 2. Results and Discussion

### 2.1. Extraction Process

To determine the optimal extraction conditions for PAR and other bioactive compounds from *T. parthenium*, two extraction techniques were compared: conventional ME and novel UE. Distilled water and two aqueous-ethanol solutions (50% and 70% *v*/*v*) were evaluated as extraction media. Each solvent was tested both in the absence and presence of 0.5% (*w*/*w*) CD, resulting in 12 combinations of extraction conditions. Extraction efficiency was assessed based on PAR yield, TPC, and TFC. The results are summarized in Table 1.

The results show that water provided the lowest PAR, TPC, and TFC values, indicating its limited suitability as an extraction solvent. This can be attributed to the low solubility of lipophilic PAR in highly polar solvents. Accordingly, water-based extraction was excluded from further optimization. In contrast, ethanol solution markedly improved extraction efficiency, likely due to reduced solvent polarity and increased mass transfer of bioactive metabolites. Only minor differences in PAR yield, TPC, and TFC were observed between 50% (*v*/*v*) and 70% (*v*/*v*) ethanol solutions. Therefore, 50% (*v*/*v*) ethanol was selected for further investigation due to its comparable efficiency, lower organic solvent consumption, reduced cost, and more favorable environmental profile.

The addition of CD increased PAR yield under both extraction techniques, while slight improvements in TPC and TFC were also observed. This enhancement can be attributed to improved solubilization of poorly water-soluble molecules, such as sesquiterpene lactones, through inclusion complex formation within hydrophobic CD cavities, as well as non-inclusion interactions such as hydrogen bonding between their external hydroxyl groups [23]. Recent evidence also suggests that CDs may form soluble aggregates or nanoscale structures that behave similarly to micelles, facilitating the transfer of hydrophobic phytochemicals from the plant matrix into the extraction solvent. Moreover, CD interactions with plant cell wall components can weaken matrix–solute interactions and enhance mass transfer, especially when combined with UE [23,24].

Compared with conventional ME, UAE improved the extraction efficiency, particularly in the presence of CD, while reducing the extraction time. These improvements can be attributed to ultrasound-induced acoustic cavitation, which involves the formation, growth, and subsequent implosive collapse of microbubbles within the extraction medium. Acoustic cavitation promotes the disruption of plant cell walls, enhances solvent penetration into the plant matrix, reduces diffusion resistance, increases mass transfer between the plant material and the extraction solvent, and accelerates extraction kinetics, thereby facilitating the release of intracellular bioactive compounds [25]. These combined mechanisms enable more efficient recovery of PAR and other phytochemicals under mild temperature conditions, thus minimizing the thermal degradation of heat-sensitive compounds. Among the tested conditions, UE performed using 50% (*v*/*v*) ethanol with the addition of CD provided the highest PAR yield of 6.30 mg/g. The superior performance of UE is consistent with previous findings [26] showing that intensified extraction techniques can overcome the limitations of conventional maceration, such as prolonged extraction times, higher solvent consumption, and frequently lower recovery of targeted phytochemicals.

Although UE cavitation promotes cell wall disruption and enhances the release of intracellular bioactive compounds, the applied extraction conditions were sufficiently mild to minimize possible degradation. The extraction temperature was maintained at 25 °C, limiting thermal stress and preserving the stability of thermolabile phytochemicals. UE has been shown to improve the extraction of phenolic and flavonoid compounds from plant matrices by enhancing matrix disruption and solvent accessibility, resulting in a TPC of 75.76 mg GAE/g and a TFC of 28.59 mg CE/g. Similarly, Zengin et al. [27] demonstrated that the choice of extraction technique directly influences both the chemical profile and the overall extract yield. According to their results, the application of UE resulted in significantly high levels of total phenolics (45.33 mg GAE/g extract) and flavonoids (48.49 mg rutin equivalent/g extract) [27].

Based on the presented results, 50% (*v*/*v*) ethanol with CD and UE was selected as the optimal extraction approach for subsequent experiments and for the preparation of the extract for the encapsulation process.

Based on optimization of the extraction process, 50:50 (*v*/*v*) ethanol (under the solid–solvent ratio of 1:30) was chosen as the optimal extraction condition for feverfew. The optimal extract, rich in bioactive compounds such as PAR, total polyphenolics and flavonoids, was prepared according to the principles of green chemistry using ethanol–water as a solvent and without the use of high temperatures, employing the green and innovative extraction method, UE, with CD addition.

### 2.2. Encapsulation and Characterization of Spray-Dried Powders

The spray-drying technique was employed to encapsulate and preserve bioactive compounds from feverfew extract, improve the chemical stability of the liquid extract and minimize the degradation of sensitive polyphenolic and flavonoid constituents, particularly the sesquiterpene lactone PAR, while maintaining high process efficiency. The obtained powders were characterized through their technological, physicochemical, chemical, and biological properties.

#### 2.2.1. Technological Properties of Powders

##### Spray-Drying Process Yield

Process yield is an important indicator of the efficiency of converting a liquid feed into a dry powder, and it is closely related to the technological feasibility of pharmaceutical and nutraceutical applications [28]. A yield above approximately 50% is generally considered satisfactory for industrial spray-drying operations, as it reflects efficient powder recovery and limited product loss within the drying chamber [22,29].

In the present study, the spray-drying yield increased from 47.27% for FWE to 65.28% for FWE+CD, demonstrating that the inclusion of CD as a carrier substantially improved powder production. This effect may be attributed to its dual role as a carrier and technological excipient. Through non-covalent interactions and inclusion complex formation with PAR and other bioactive constituents [10,30,31], CD may enhance droplet stability, reduce surface migration of low-molecular-weight compounds, decrease particle stickiness, and limit adhesion to the drying chamber walls. This phenomenon has been reported in a study showing that β-cyclodextrin or its derivatives enhance process yield compared to non-encapsulated feeds or conventional carriers alone [10]. In addition, CD increased the solid content and modified the rheological properties of the feed solution, promoting faster surface crust formation on atomized droplets and the formation of a more stable particle matrix during atomization and drying and reducing particle stickiness and their tendency to adhere to the spray-dryer surfaces, a common cause of product loss. Similar improvements in spray-drying yield have been reported for β-cyclodextrin and its derivatives [11]. The lower yield of unencapsulated FWE was likely caused by the absence of a protective carrier matrix and the higher tendency of low-molecular-weight compounds to adhere to the dryer surfaces. The enhanced process yield observed for FWE+CD confirms the beneficial role of CD in improving spray-drying efficiency by facilitating droplet drying and reducing wall deposition.

##### Moisture Content

The moisture content of the powders differed significantly between the formulations, decreasing from 8.30% in the carrier-free FWE to 6.94% in FWE+CD (Table 2). The incorporation of CD significantly reduced residual moisture, indicating improved drying efficiency. Compared with the carrier-free extract, the CD-based system provided a protective matrix that limited moisture uptake and improved resistance to environmental humidity.

This effect may be attributed to the increased total solids content and modified feed composition, which can facilitate faster particle formation during spray drying. In contrast, carrier-free powder is more prone to moisture due to the presence of low-molecular-weight compounds, which contribute to stickiness and hygroscopicity [32,33]. A similar effect of CD as a carrier on reducing residual moisture and improving resistance to atmospheric water uptake has been reported previously [10,17]. Therefore, the lower moisture content observed for FWE+CD supported the role of CD not only as an encapsulation agent but as a critical technological excipient that significantly improves the quality attributes and physicochemical stability of spray-dried feverfew extract.

The effects of residual moisture content are particularly important for spray-dried plant extracts, where low-molecular-weight compounds may increase hygroscopicity and promote stickiness during storage.

##### Bulk and Tapped Densities

Bulk and tapped densities are important technological parameters for powders intended for pharmaceutical [34] and food processing [35], as they affect handling, packaging, capsule filling, tablet formulation, and dermo-cosmetic formulation.

**Scheme 1:**
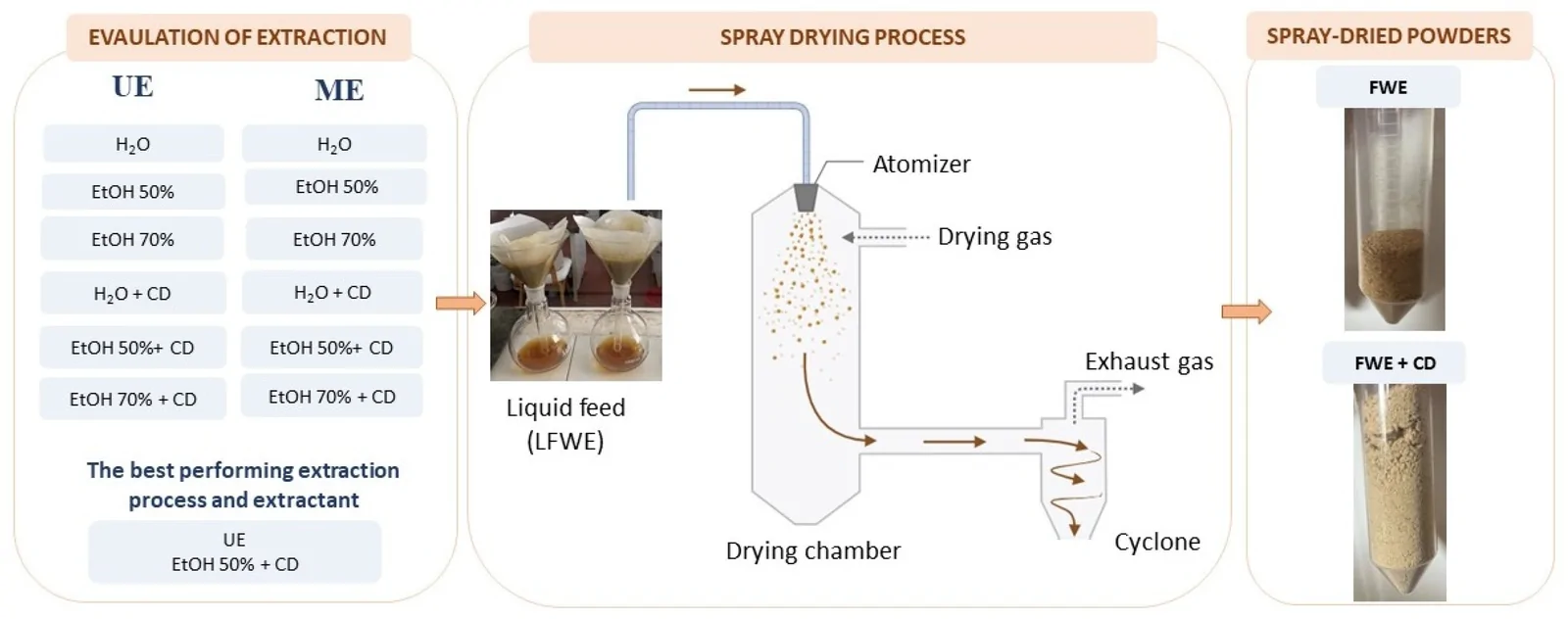
Extractants used for ultrasound-assisted extraction (UE), maceration extraction (ME), and spray-drying encapsulation of feverfew extract. The control powder sample (FWE) was prepared without the addition of CD. Hydroxypropyl-β-cyclodextrin (CD), spray-dried feverfew extract (FWE) (the control powder sample prepared without the addition of CD), spray-dried feverfew extract with CD (FWE+CD).

In this study, FWE exhibited a bulk density of 0.46 g/mL, whereas FWE+CD showed a lower value of 0.26 g/mL (Table 2). The approximately two-fold reduction in bulk density after CD incorporation indicates the formation of a lighter, more voluminous powder bed (Scheme 1), which is typical for spray-dried inclusion systems due to the amorphous structure and lower density of CD matrices. This behavior may also reflect differences in particle morphology, surface characteristics, and interparticle interactions introduced by CD. Similar bulk density ranges have been reported for spray-dried powders [10]. The greater capacity of FWE+CD reflects enhanced particle rearrangement within the porous CD-based matrix.

Flowability assessment based on the CI and HR showed that both powders have technologically acceptable flow properties. FWE exhibited excellent flowability, with a CI of 6.67 and an HR of 1.07, while FWE+CD showed good flowability, with a CI of 13.49% and an HR of 1.16. The higher CI and HR values of FWE+CD indicate moderately enhanced interparticle cohesion and compressibility, which can be attributed to the amorphous character and the partial hygroscopicity of the CD. Similar trends have been reported for spray-dried phytopharmaceutical systems containing CDs, where inclusion complex formation improves stability but slightly reduces flow performance [10]. Nevertheless, both formulations remain within technologically acceptable limits for pharmaceutical and food processing, confirming their suitability for further formulation steps, without the need for additional flow-enhancing excipients.

Rehydration properties were assessed as the time required for complete dispersion in water. A pronounced difference was observed between the formulations. Namely, FWE showed rapid rehydration (7.66 s), whereas FWE+CD required a significantly longer time (52.40 s) (Table 2). The rapid rehydration of FWE is associated with superficial wetting of compact particles, without structural relaxation. Conversely, the prolonged rehydration of FWE+CD likely reflects the presence of a more organized CD-based matrix, where water penetration, matrix relaxation, and dispersion of the inclusion complex govern the reconstruction process. A similar behavior was found in the reconstitution kinetics of spray-dried pomegranate powders [22]. Thus, although CD incorporation increases rehydration time, it may contribute to controlled and homogeneous dispersion, which is essential for consistent formulation performance.

pH values of both powders were narrowly distributed, with FWE showing a mean pH value of 5.08 and FWE+CD samples a value of 5.16 (Table 2). The slight increase in pH after CD incorporation may indicate partial interactions with the extract’s acidic constituents, reducing their availability in aqueous solution. Both samples exhibited mild natural acidity with pH values near 5, which is favorable for maintaining physicochemical stability and may be suitable for dermal and oral application formulations.

While CD incorporation slightly prolonged rehydration time, it improved structural dispersion characteristics and produced a slight buffering effect. These results support the role of CDs as multifunctional excipients influencing both the technological performance and physicochemical stability of plant-derived powder formulations.

#### 2.2.2. Physicochemical Characterization of the Powders

##### Powder Composition Analysis by FTIR Analysis

**Figure 1:**
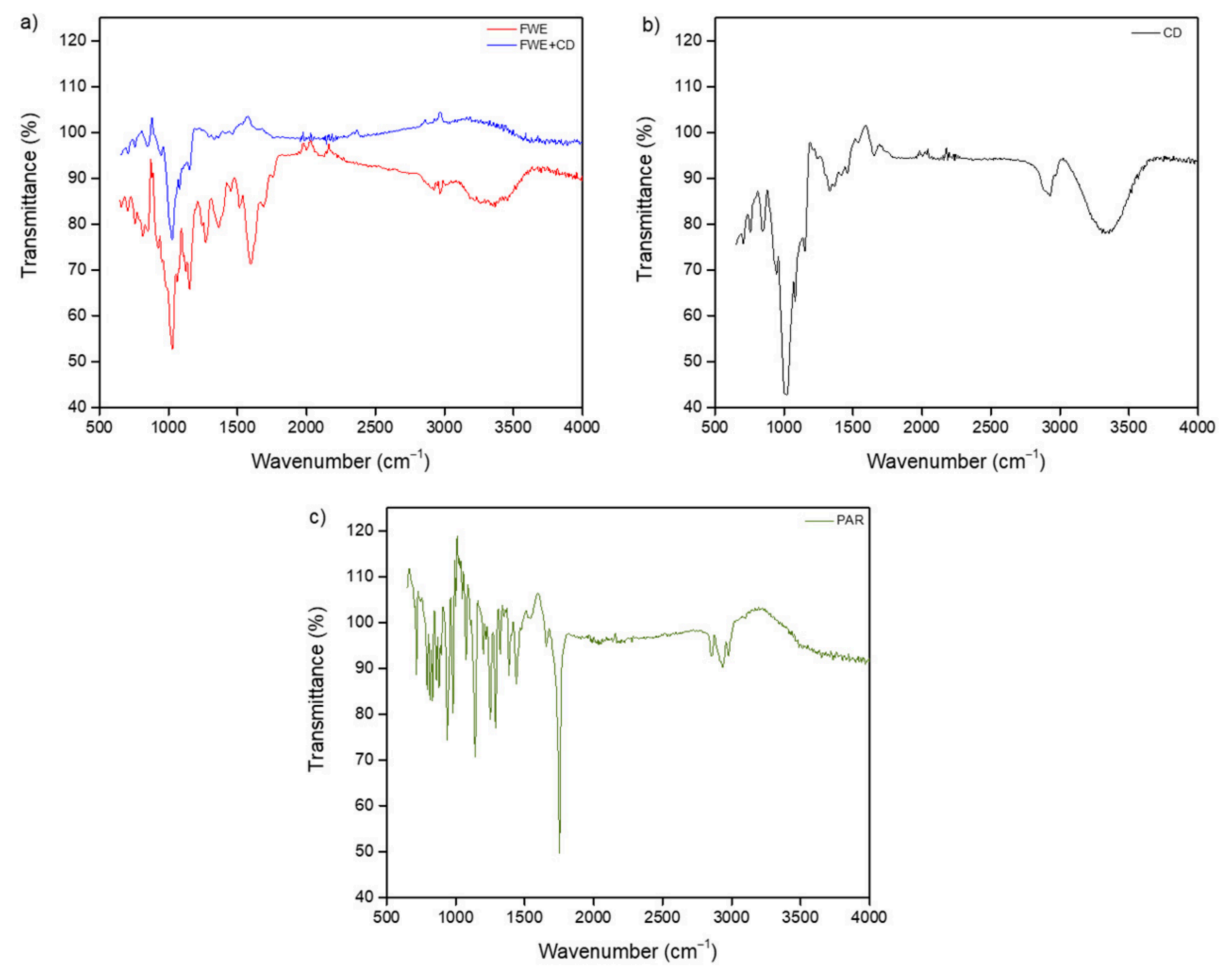
FTIR spectra of (**a**) spray-dried feverfew extract (FWE), the encapsulated sample (FWE+CD), (**b**) cyclodextrin (CD), and (**c**) pure parthenolide (PAR).

FTIR spectroscopy was used to evaluate the chemical characteristics of FWE, CD, pure PAR, and FWE+CD, as well as potential interactions formed during encapsulation (Figure 1). The FTIR spectrum of CD (Figure 1b) showed a broad band at 3200–3400 cm^−1^, attributed to O–H stretching vibrations of hydroxyl groups; a band around 2920 cm^−1^ corresponding to C–H stretching; and characteristic C–O and C–O–C stretching bands in the 1150–1000 cm^−1^ region, typical of glucopyranose units in CD [24]. The spectrum of FWE (Figure 1a) displayed a broad O–H stretching band at 3350 cm^−1^, associated with phenolic and other hydroxyl-containing compounds, together with bands observed around aliphatic C–H stretching at 2900–2800 cm^−1^. The absorption bands at 1750 cm^−1^ are attributed to C=O stretching of lactone groups, characteristic of sesquiterpene lactones such as PAR, the major bioactive component of feverfew [36].

The spectrum of FWE+CD (Figure 1a) retained the main absorption bands of both the carrier and extract, indicating that the chemical structure of the extract components remained unchanged after the spray-drying encapsulation process. However, slight band shifts and broadening, particularly in the O–H stretching region and within the 1000–1100 cm^−1^ range, suggest hydrogen bonding and physical interactions between CD and the extract constituents. The absence of new absorption bands further indicates that encapsulation occurred via non-covalent interactions within the CD cavity rather than chemical modification. Comparison with pure PAR (Figure 1c) confirmed the relevance of the characteristic lactone carbonyl band (1750 cm^−1^). This signal is less pronounced in FWE and is no longer clearly visible in FWE+CD, most likely due to masking by the CD matrix and the partial inclusion or physical entrapment of PAR within the CD cavity. [31,37]. Such spectral changes are typical in CD inclusion complexes, where guest molecules are stabilized primarily by hydrophobic interactions and hydrogen bonding [38]. Therefore, the FTIR results suggest that PAR remains present in the encapsulated system and is physically entrapped or partially included within the CD matrix, which may contribute to improved stability and protection of the bioactive compound.

##### Particle Size Analysis of the Powders

**Figure 2:**
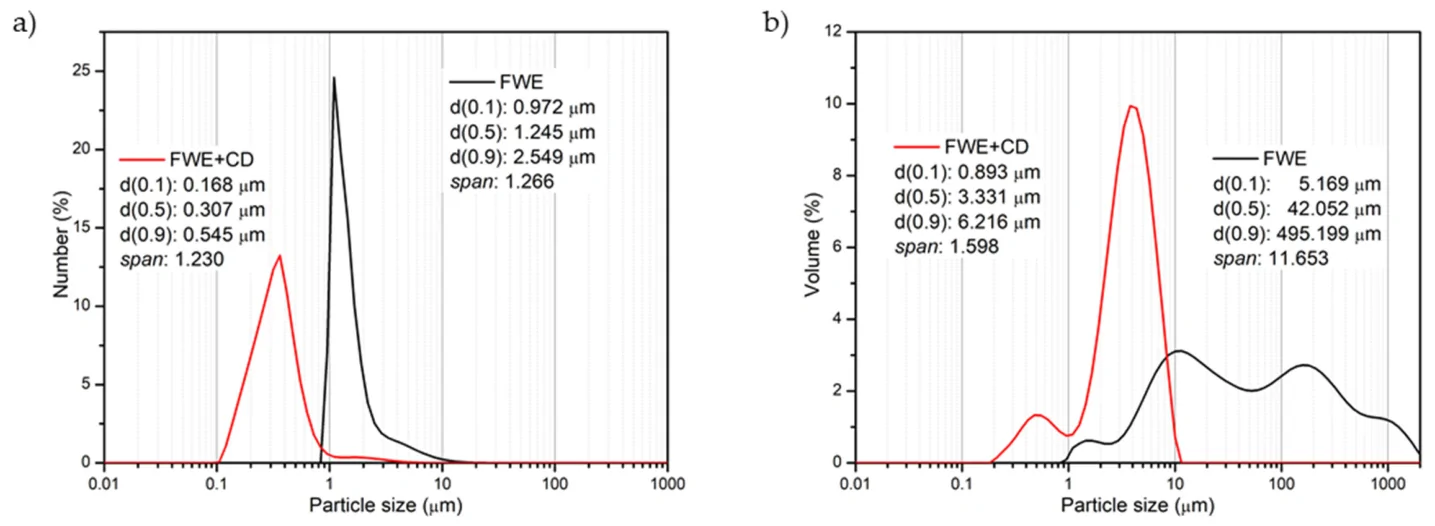
Particle size distribution of spray-dried feverfew extract (FWE) and FWE encapsulated within 2-hydroxypropyl-β-cyclodextrin (FWE+CD) powder: (**a**) number-based and (**b**) volume-based analyses.

The particle size distribution of FWE and FWE+CD was evaluated using both number-based and volume-based analyses (Figure 2). Since volume distribution more accurately reflects the contribution of larger particles to the overall powder system, it was primarily used for interpretation. The number distribution was used to describe the presence of submicron particles. Number-based analysis showed that FWE+CD consisted mainly of submicron particles, with d (0.1), d (0.5), and d (0.9) values of 0.168, 0.307, and 0.545 μm (SPAN = 1.230), while FWE exhibited larger particles with values of 0.972, 1.245, and 2.549 μm (SPAN = 1.266), respectively. This indicates that CD incorporation shifted the distribution toward smaller particle sizes. The difference between the samples was more evident in the volume-based particle size distribution. FWE exhibited significantly larger particle sizes, with d (0.1), d (0.5), and d (0.9) values of 5.169, 42.052, and 495.199 μm, respectively, along with a high SPAN value (11.653), indicating a highly polydisperse system and the presence of large particle aggregates. In contrast, FWE+CD showed substantially smaller and more uniformly distributed particles, with d (0.1), d (0.5), and d (0.9) values of 0.893, 3.331, and 6.216 μm, and a lower SPAN value of 1.598. The discrepancy between number- and volume-based distributions for FWE and FWE+CD suggests that a small number of large agglomerates contribute disproportionately to the particle volume, representing only a minor fraction of the particle population. Such aggregation is commonly observed in spray-dried plant extracts due to the sticky amorphous components, such as sugars and organic acids, which can promote particle agglomeration during drying and powder collection. The addition of CD clearly reduced aggregation and improved particle size uniformity, mostly by modifying feed properties such as viscosity and surface tension, thereby improving atomization and promoting the formation of smaller droplets during spray drying [10,31]. The median particle diameter of FWE+CD, 3.331 μm, is consistent with previously reported CD-based spray-dried encapsulation systems, which typically show particle sizes in the low micrometer range [27,31,39]. The combined number- and volume-based analysis confirmed that CD encapsulation reduced particle size and markedly improved particle size uniformity, resulting in a more homogeneous powder suitable for stabilization and delivery. The smaller and more uniform particles of FWE+CD suggest more efficient encapsulation and better incorporation of the extract into the carrier matrix. This structural organization may limit the exposure of sensitive compounds, particularly PAR, to oxygen, light, and moisture, thereby improving storage stability. Furthermore, the reduced particle size and improved dispersibility of FWE+CD may favor dissolution and release behavior, with potential benefits for the bioavailability of PAR and other phytochemicals present in FWE.

##### Differential Scanning Calorimetry Analysis

**Figure 3:**
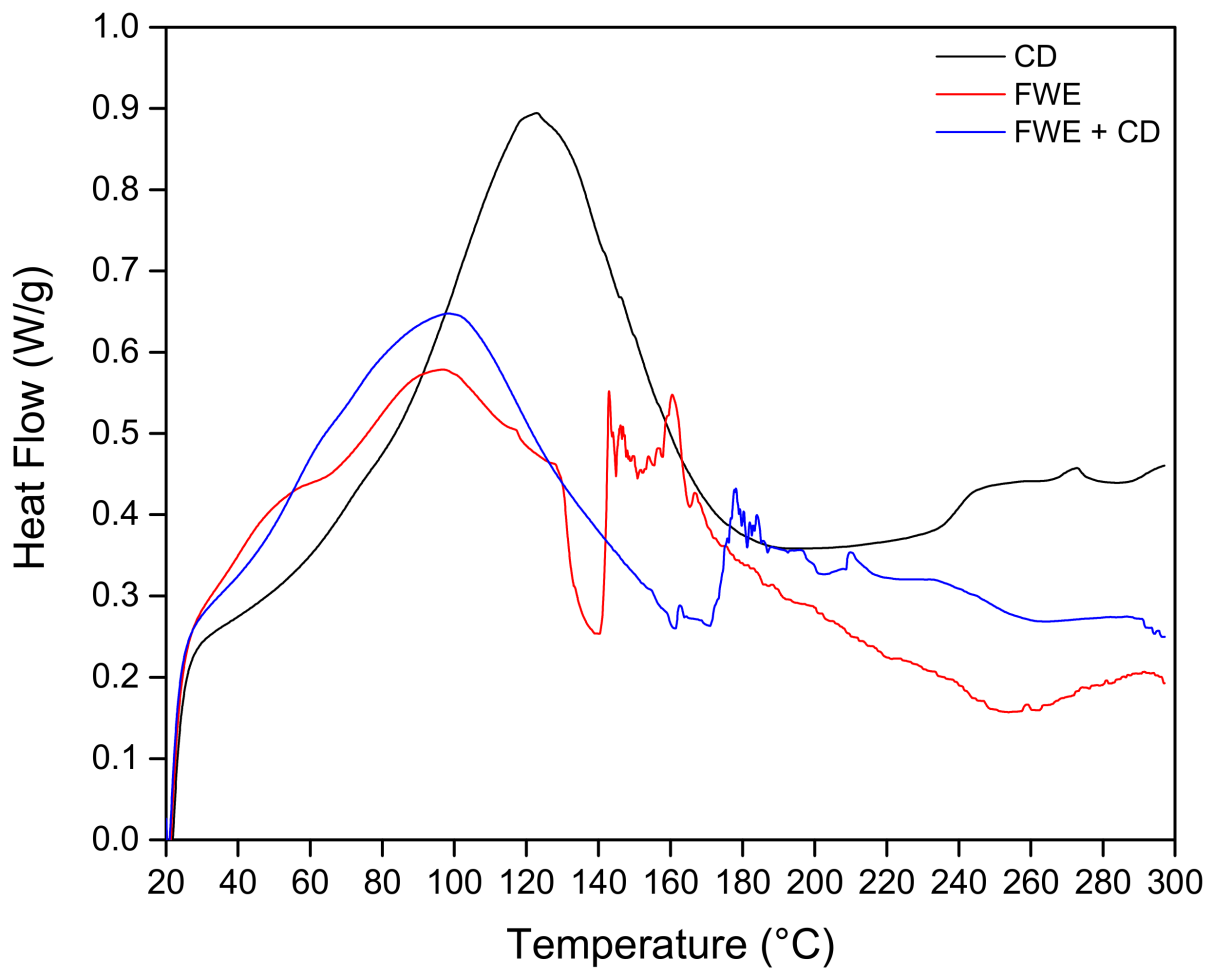
DSC thermograms of 2-hydroxypropyl-β-cyclodextrin (CD) and spray-dried powders of feverfew extract (FWE) and FWE encapsulated within CD (FWE+CD).

DSC evaluated the thermal properties of spray-dried FWE, FWE+CD, and the carrier, and the corresponding thermograms are presented in Figure 3, with enthalpy change values and peak temperatures (Table 3). The observed thermal transitions were mainly associated with dehydration and thermal degradation of the analyzed materials, providing insight into the stability of the encapsulated bioactives under thermal stress.

The DSC thermogram of FWE showed a temperature change range from 21.19 to 297.14 °C, exhibiting a pronounced endothermic peak with a maximum around 90 °C, which can be attributed to the evaporation of bound water and the initial thermal degradation of thermolabile extract components. A second broader thermal event with a temperature peak of 160 °C was also observed, probably corresponding to the decomposition of organic constituents such as phenolic compounds, sesquiterpene lactones, and other phytochemicals present in FWE. The carrier CD exhibited a temperature change degradation from 21 to 272.79 °C, with a broad endothermic peak with a maximum around 119 °C, commonly attributed to the release of water molecules from the CD cavity and dehydration of the amorphous carbohydrate structure [40,41]. In contrast, for FWE+CD, the observed temperature transitions shifted, occurring between 36.02 and 297.15 °C, with a main endothermic peak with a maximum around 98 °C, positioned between those of the pure extract and the carrier. This shift, together with changes in peak intensity and enthalpy, suggests interactions between the FWE components and the CD matrix. The increased enthalpy observed for FWE+CD indicates a higher energy requirement for thermal degradation, suggesting improved thermal stability of the encapsulated extract compared to the unencapsulated one. This behavior is commonly interpreted as evidence of encapsulation or inclusion complex formation, with the guest molecules partially entrapped within the CD cavity, reducing molecular mobility and protecting thermolabile bioactive compounds from premature degradation [42,43,44].

The DSC results confirm that carrier incorporation provides a protective environment for FWE components, potentially improving their stability during processing and storage.

##### X-Ray Diffraction Analysis

**Figure 4:**
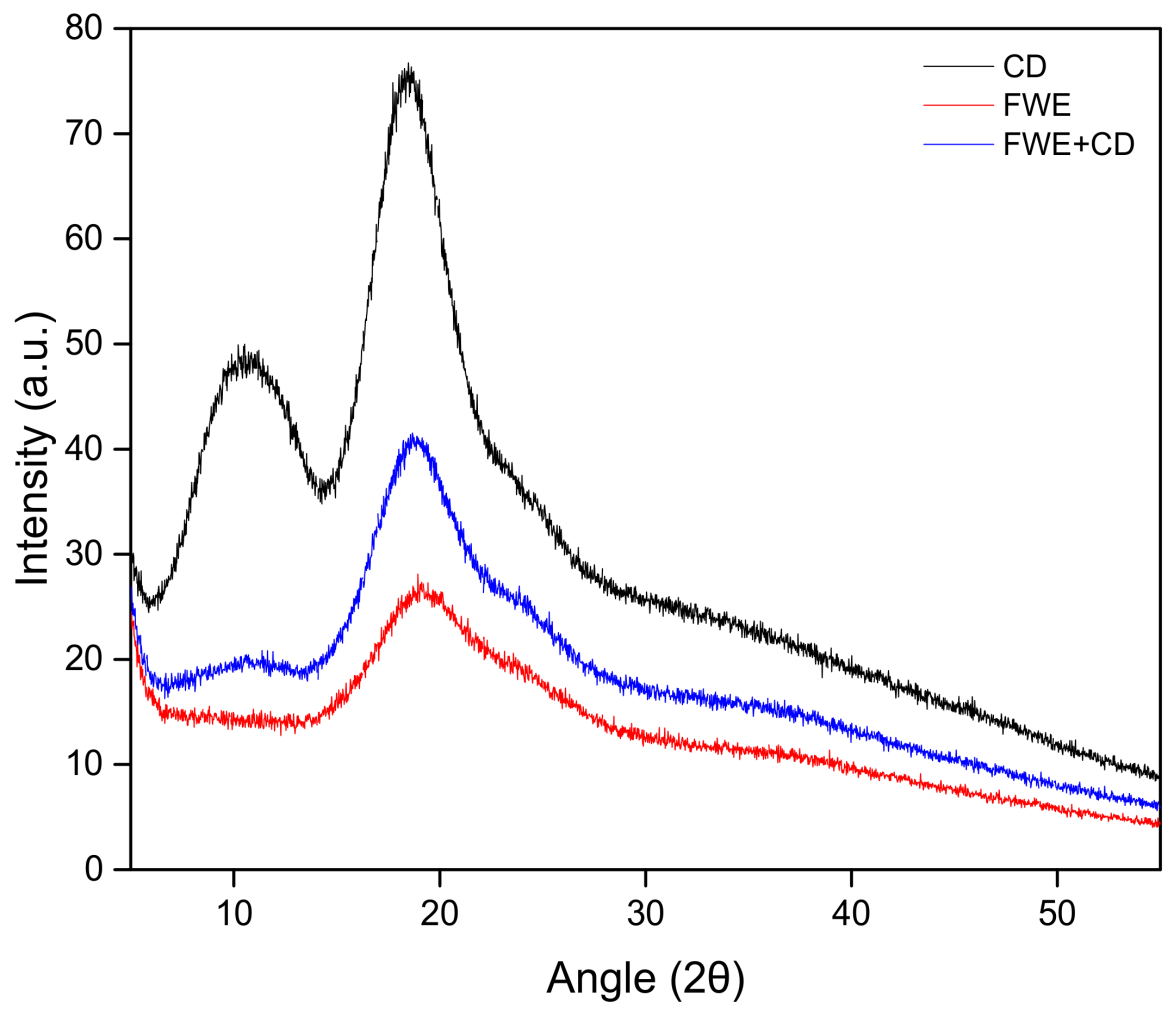
XRD diffractograms of *Tanacetum parthenium* L., 2-hydroxypropyl-β-cyclodextrin (CD), spray-dried feverfew extract (FWE), and FWE encapsulated within CD (FWE+CD).

XRD analysis was performed to evaluate the structural characteristics and crystallinity profile of plain feverfew plant material, spray-dried FWE, and the encapsulated powder, FWE+CD, as well as the effect of carrier addition on the solid-state structure (Figure 4). The dried feverfew plant exhibited several relatively sharp diffraction peaks, which may be associated with native crystalline compounds, such as minerals, inorganic salts, or other native crystalline compounds present in the plant matrix [45]. After spray drying, FWE exhibited reduced peak intensity and partial peak broadening, suggesting decreased crystallinity and increased amorphization. However, the main diffraction maxima remained at similar 20 positions, indicating that spray drying did not substantially alter the extract’s chemical composition. The observed reduction in crystallinity is typical for spray-dried materials, where rapid solvent evaporation prevents molecular rearrangement and crystal formation, leading to a more amorphous structure [14,46].

FWE+CD demonstrated a further increase in amorphous characteristics, probably due to the presence of the spray-dried carrier. In this diffractogram, the characteristic peaks of FWE were significantly reduced or masked, while a broad amorphous group became dominant. Although CDs are crystalline in their native form, spray drying can promote their transition to a more amorphous structure. The suppression of FWE diffraction peaks suggests that the extract components were molecularly dispersed within the CD matrix, possibly through partial inclusion complex formation and solid-state dispersion. This indicates effective encapsulation of extract components within the CD structure. Similar structural changes, including reduced crystallinity and amorphous broadening, have been reported for spray-dried plant extract–CD systems [47] and CD complexes with phytochemicals such as polyphenols and essential oils [24,31,48].

The XRD analyses are consistent with the DSC results, indicating that the increased amorphous character of the spray-dried samples, particularly FWE+CD, correlates with improved thermal stability. The reduced crystallinity suggests molecular dispersion of the extract components within the CD matrix, which may contribute to improved protection of bioactive compounds.

##### Scanning Electron Microscopy and Energy-Dispersive X-Ray Spectroscopy Analyses of Spray-Dried Powders

**Figure 5:**
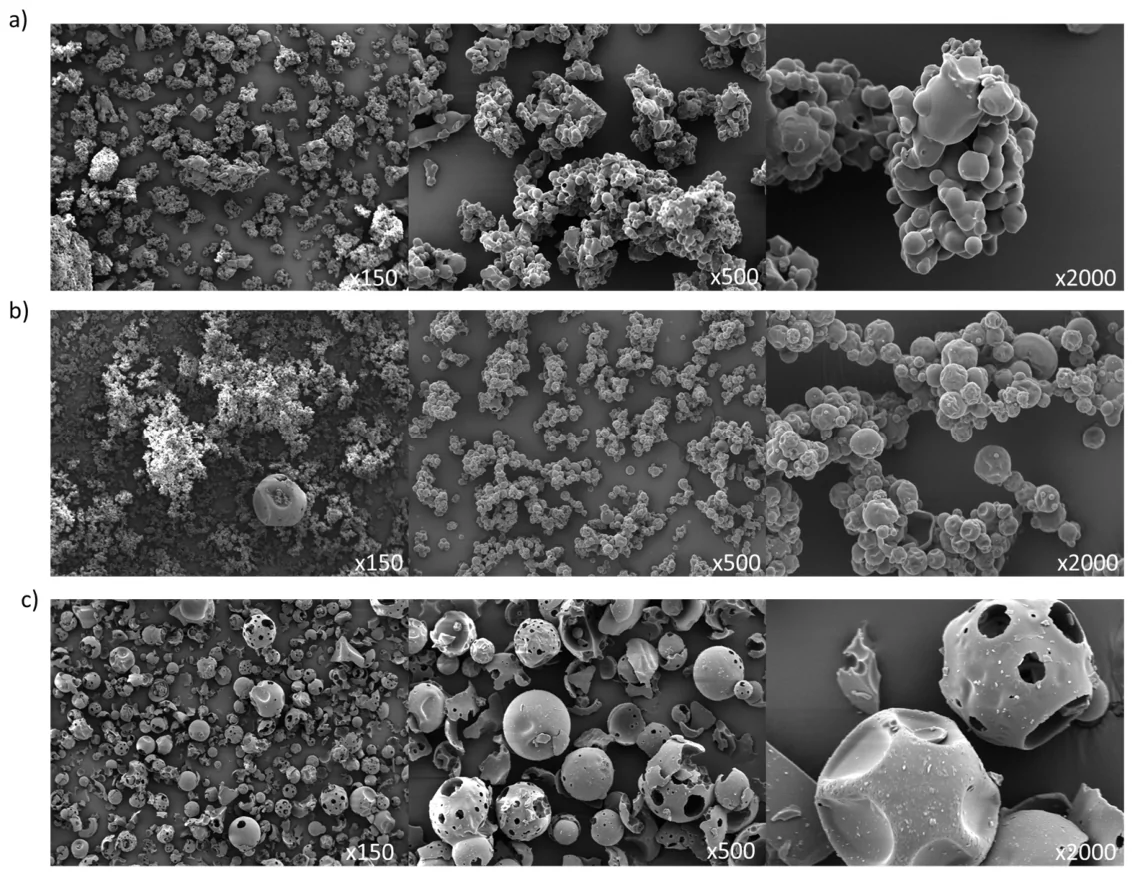
Scanning electron microscopy images of (**a**) 2-hydroxypropyl-β-cyclodextrin (CD), (**b**) feverfew extract (FWE), and (**c**) feverfew extract encapsulated with CD (FWE+CD); magnifications of 150, 500, and 2000 times.

The surface morphology and microstructural properties of the spray-dried powders were evaluated by SEM (Figure 5a–c), supported by EDS analysis (Figure S1). EDS analysis showed that all samples (FWE, FWE+CD, and CD) were mainly composed of carbon and oxygen, confirming their predominantly organic nature. In addition, FWE and FWE+CD contained minor amounts of mineral elements, including potassium, chlorine, phosphorus, sulfur, calcium, magnesium, and sodium, which are naturally present in plant tissues [49].

SEM analysis revealed clear morphological differences among FWE, FWE+CD, and CD, reflecting the critical role of formulation composition in particle formation during spray drying. The morphology of CD showed relatively uniform, amorphous particles with smooth to slightly wrinkled surfaces (Figure 5a), consistent with previously reported spray-dried CD powders [48,50]. In contrast, FWE exhibited highly irregular, collapsed particles with a broad size distribution and rough surface (Figure 5b), indicating particle shrinking and structural collapse during spray drying. Such morphology is typical in spray-dried plant extracts without adequate carrier protection, where sticky amorphous constituents promote deformation and agglomeration [51,52].

The FWE+CD formulation showed significantly improved morphology, with predominantly spherical particles, more uniform size distribution, and smoother surfaces (Figure 5c). Although slight surface wrinkling was observed, the particles retained their structural integrity without pronounced collapse. These improvements can be attributed to the matrix-forming ability of CD, which reduces stickiness, supports shell formation during droplet drying, and promotes more efficient extract encapsulation [10,31,32,46]. Inclusion complex formation between CD and hydrophobic constituents such as PAR may contribute to a more homogeneous distribution of bioactives within the matrix, further stabilizing the particle structure [24]. Analogous mechanisms have been described in spray-dried systems containing polyphenols and essential oil components, where CD incorporation leads to improved encapsulation efficiency and particle morphology [10,43]. When compared to other commonly used carriers (e.g., maltodextrin or gum arabic), CDs often produce particles with slightly more wrinkled surfaces but superior molecular-level stabilization due to host–guest interactions [53,54]. From a technological standpoint, the irregular and collapsed morphology of FWE is typically associated with poor powder flowability, higher hygroscopicity, and moisture. On the other hand, the more spherical and structurally stable particles observed in FWE+CD are indicative of improved powder properties, including enhanced flow behavior, reduced agglomeration, and better reconstitution in aqueous systems, which are in agreement with previous results. Namely, SEM analysis confirms that CD plays a crucial role in modulating particle formation and improving the physical quality of spray-dried feverfew formulations [24].

The amorphous morphology observed in SEM micrographs, particularly for FWE+CD, is consistent with DSC and XRD analyses, supporting the formation of predominantly amorphous systems and partial inclusion complexation. The correlation between particle integrity observed by SEM and the absence of crystalline phases further supports the successful molecular encapsulation and stabilization of feverfew bioactives within the CD matrix.

#### 2.2.3. Chemical Analysis of Spray-Dried Powders

The prepared powder formulations (FWE and FWE+CD) were evaluated for PAR content, TPC, and TFC. PAR is selected as the key marker compound because it is the major bioactive sesquiterpene lactone compound in *T. parthenium* and is commonly used for feverfew-based formulation standardization [1,55]. The European Pharmacopoeia [56] specifies a minimum PAR content of 0.2% in the herbal drug, while standardized extracts are typically adjusted within 0.2–0.8% (approximately 2–8 mg/g), depending on plant part and processing conditions [1]. In addition to sesquiterpene lactones, feverfew extracts contain a structurally diverse phenolic fraction, including hydroxycinnamic acids and flavonoids [55,57]. Polyphenolic compounds and flavonoids that are also recognized as contributors to their biological properties. Therefore, the total phenolic content (TPC) and total flavonoid content (TFC) were determined as complementary phytochemical parameters and were intended to provide a broader characterization of the extract composition and to support the interpretation of the biological activity of the obtained microencapsulates.

The determined PAR concentration was 7.628 mg/g in FWE and 5.955 mg/g in FWE+CD. The TPC values reached 117.51 mg GAE/g for FWE and 110.45 mg GAE/g for FWE+CD, while TFC was 50.30 and 41.60 mg CE/g, respectively. Comparable retention of polyphenolic compounds has been reported for spray-dried plant extracts, where phenolic compounds are well preserved due to rapid solvent removal and limited thermal degradation during atomization [31]. The slightly lower PAR, TPC, and TFC values observed in the CD-containing formulation may be attributed to dilution effects caused by CD addition, as well as to possible host–guest interactions between CD and phenolic molecules. Such an interaction may alter compound solubility and physicochemical behavior, potentially affecting their analytical accessibility and quantification [58].

Comparative studies on CD-based encapsulation systems have demonstrated that inclusion complex formation can promote molecular-level dispersion of bioactive compounds within polymeric or carbohydrate matrices. Specifically, it may alter the extractability and analytical responses of phenolic constituents. Similar structural and compositional effects have been reported for spray-dried CD microencapsulation of phytochemicals, where host–guest interactions contribute to improved stability and modified physicochemical behavior of the encapsulated actives [31].

These findings indicate that the applied extraction and drying procedures successfully preserved the characteristic phytochemical profile and provided a robust baseline for subsequent stability studies. Spray drying has been extensively used for the encapsulation of thermolabile bioactives, such as curcumin, leading to improved stability and protection during processing. In particular, microencapsulation has been shown to enhance the heat stability of curcumin while preserving its antioxidant activity [59].

#### 2.2.4. Biological Activities

##### Cytotoxicity Properties of FWE and the Encapsulate (FWE+CD) in Cell-Based Assays

The cytotoxicity of FWE and FWE+CD was evaluated in HaCaT keratinocytes using the MTT assay. Specifically, cell viability was evaluated using the MTT assay, a well-established colorimetric method widely used for the assessment of cytotoxic effects of various compounds. Although alternative methods, such as the CCK-8 assay, provide certain advantages, including increased sensitivity and a simpler workflow, the MTT assay remains a widely accepted and validated approach for evaluating cell metabolic activity and cytotoxic responses. The MTT assay was selected due to its high reproducibility, reliability, and compatibility with the experimental conditions used in this study. Additionally, plain CD and PAR were also tested.

**Figure 6:**
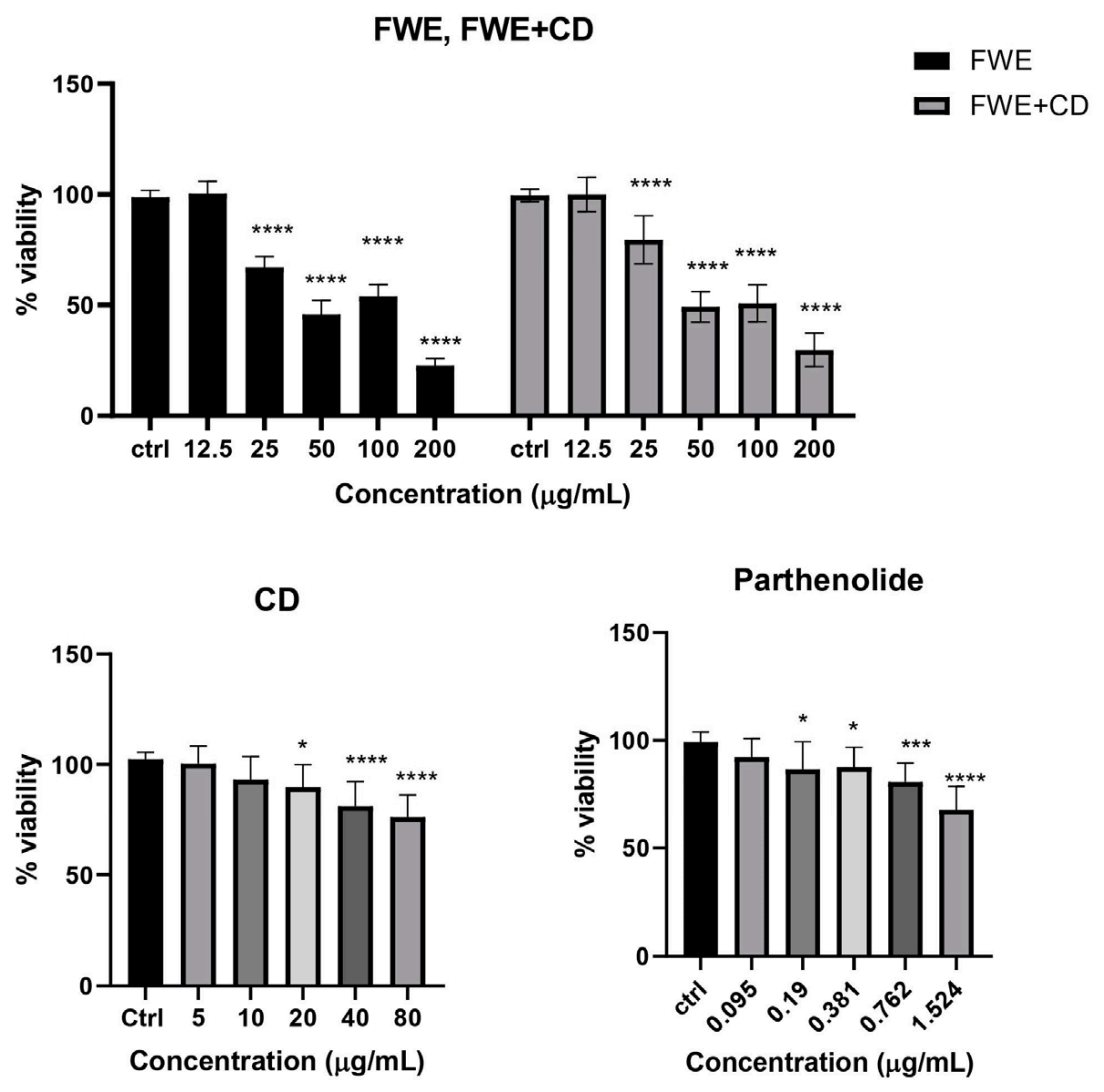
Cytotoxicity of the feverfew extract (FWE) and the extract in 2-hydroxypropyl-β-cyclodextrin (FWE+CD) in a range of concentrations (12.5–200 µg/mL), as well as CD (5–80 µg/mL) and parthenolide (0.095–1.524 µg/mL), determined by the MTT assay in the HaCaT cells. Data are expressed as mean ± standard error of the mean relative to the unexposed control; one-way analysis of variance (ANOVA) with Tukey’s multiple comparison post hoc test (n = 6, * *p* < 0.05, *** *p* < 0.001, **** *p* < 0.0001).

The cytotoxic effects of the mentioned powders were determined in the HaCaT cell line (keratinocytes) using the MTT assay, confirming a favorable safety profile of both spray-dried formulations only at the lowest tested concentration. As shown in Figure 6, FWE and FWE+CD exhibited concentration-dependent cell viability, with a favorable cytocompatibility profile observed only at the lowest tested concentration.

FWE causes a significant reduction in viability from 25 µg/mL onward, reaching approximately 20–30% cell viability at 200 µg/mL, indicating that the FWE contains bioactive constituents that markedly impair keratinocyte viability. In comparison, FWE+CD showed a similar but slightly attenuated cytotoxic profile, suggesting that CD complexation may modulate the bioavailability and/or cellular uptake of active compounds. These results should be interpreted considering the dilution of FWE within the carrier-containing formulation, which also contributed positively to the powder characteristics described above.

CD alone exhibited relatively low cytotoxicity, with only a moderate decrease in cell viability at the highest tested concentrations (40–80 µg/mL), confirming its suitability as a biocompatible carrier.

PAR also reduced HaCaT viability in a dose-dependent manner, although at much lower concentrations (sub-µg/mL range). This is consistent with previous reports showing that PAR exhibits antiproliferative activity in various cell lines at low micromolar concentrations (2–4 µM) [60]. In the present study, the highest tested formulation concentration (200 µg/mL) corresponds to approximately 1.5 µg/mL of PAR, which is within or slightly below the concentration range typically associated with strong cytotoxic effects. This explains why only moderate reductions in viability were observed, rather than pronounced cytotoxicity. Although the reduction in viability was more gradual in comparison with FWE, statistically significant effects were observed at higher concentrations (≥0.381 µg/mL), with the most pronounced decrease at 1.524 µg/mL. These results indicate that PAR contributes to the cytotoxic activity of FWE, although it does not fully explain the effect of the whole extract, implying possible synergistic interactions among multiple constituents in FWE.

The FWE+CD formulation showed slightly lower cytotoxicity than FWE, which can be attributed to the protective effect of CD and the slower release of bioactive compounds. This is in line with previous reports indicating that CD inclusion complexes can reduce the apparent toxicity of lipophilic phytochemicals by modulating their bioavailability and cellular interactions [61,62]. Furthermore, the tested concentration range (12.5–200 µg/mL) is highly relevant for pharmaceutical applications, where maintaining cell viability above 70% is considered acceptable for biocompatible systems. Importantly, the concentrations of CD and PAR in the FWE+CD formulation were proportional to their actual content (e.g., at 200 µg/mL, 80 µg/mL CD and 1.5 µg/mL parthenolide), indicating that the improved cytocompatibility is not simply a consequence of reduced exposure, but rather of modified release and physicochemical behavior. These findings indicate that FWE exerts strong cytotoxic effects on HaCaT cells, which are partially modulated by CD complexation.

From an application perspective, these findings are particularly relevant for dermal formulations, as PAR-containing feverfew extracts have been associated with skin sensitization [3]. The results of this study suggest that CD encapsulation may represent an alternative strategy for improving tolerability while preserving the active compound.

##### Antioxidant Properties of FWE and FWE+CD in Cell-Based Assays

**Figure 7:**
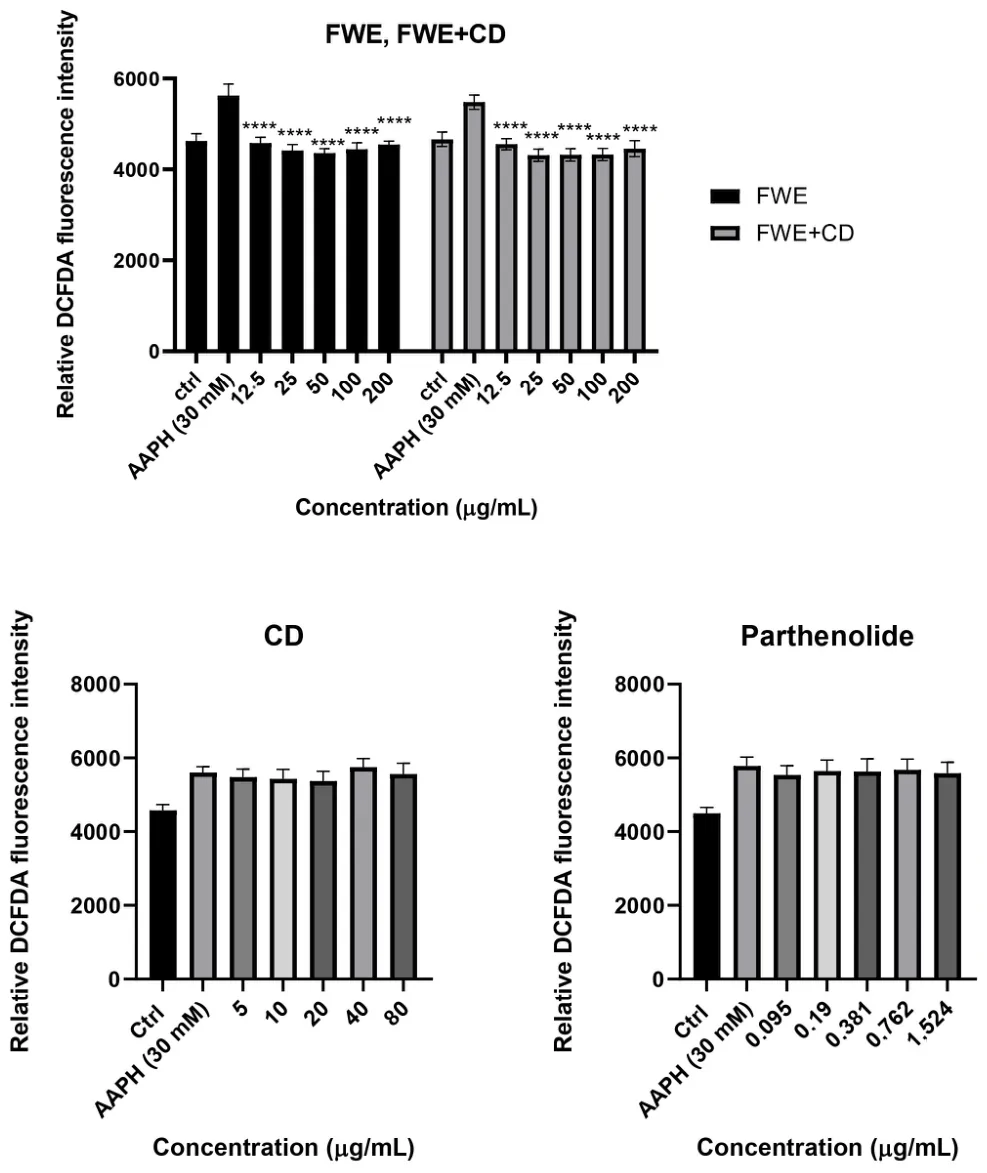
The effect of feverfew extract (FWE) and the extract in 2-hydroxypropyl-β-cyclodextrin—CD (FWE+CD) in a range of concentrations (12.5–200 µg/mL), as well as CD (5–80 µg/mL) and parthenolide (0.095–1.524 µg/mL), on intracellular reactive oxygen species scavenging capacity under conditions of AAPH-induced oxidative stress in HaCaT cells, measured by DCFDA fluorescence. The data are expressed as the mean + standard deviation (**** *p* < 0.0001 vs. AAPH) by one-way analysis of variance (ANOVA) with Tukey’s multiple comparison post hoc test, n = 6; AAPH, 2,2′-azobis(2-methylpropionamidine) dihydrochloride.

The intracellular antioxidant activity of FWE and FWE+CD, as well as CD and PAR, was evaluated using the DCFDA keratinocyte-based assay, which enables the quantification of ROS through fluorescence intensity. As shown in Figure 7, exposure to AAPH (30 mM) significantly increased intracellular ROS levels, confirming successful induction of oxidative stress.

FWE significantly reduced ROS levels at all tested concentrations (12.5–200 µg/mL), indicating pronounced antioxidant activity. This effect was not strongly dose-dependent, suggesting that even the lowest tested concentrations of FWE were sufficient to achieve maximal antioxidant activity. Similarly, the FWE+CD formulation exhibited a comparable antioxidant profile, with a consistent reduction in fluorescence intensity relative to the AAPH-treated group. In some cases, this effect was slightly more pronounced than that of FWE, which may be related to the improved stability, solubility, or cellular availability of antioxidant constituents after CD complexation.

CD alone did not reduce ROS levels, confirming its role as a biologically inert carrier in this study. Similarly, PAR did not show relevant antioxidative activity under the tested conditions, as the fluorescence intensity remained comparable to that of AAPH-treated cells. This suggests that the antioxidant effects of FWE are mainly associated with other extract constituents, particularly flavonoids and phenolic acids, rather than PAR [1,63]. This observation is consistent with previous studies indicating that feverfew’s antioxidant activity is primarily linked to its phenolic composition, while PAR is more commonly associated with anti-inflammatory effects rather than antioxidant effects [36,64]. Namely, the antioxidant capacity of feverfew extract is attributed mainly to flavonoids and phenolic acids rather than sesquiterpene lactones [1,63]. Previous studies have demonstrated that feverfew extracts exhibit moderate-to-strong antioxidant activity in spectrophotometric methods (DPPH and ABTS) and cellular assays, depending on extraction conditions and phenolic composition [1,55]. The marked intracellular reduction in ROS observed in this study suggests that the applied extraction and spray-drying conditions preserved the functional integrity of these bioactive compounds. The results indicate that FWE possesses significant antioxidant properties in HaCaT cells under oxidative stress, and that these properties are maintained or slightly enhanced upon complexation with CD. In contrast, CD alone is biologically inactive in this context, while PAR does not contribute to ROS scavenging, highlighting the complexity of the extract’s bioactivity profile.

##### Anti-Inflammatory (Membrane Stabilization) Properties of the Powders in Heat-Induced Erythrocyte Lysis

**Figure 8:**
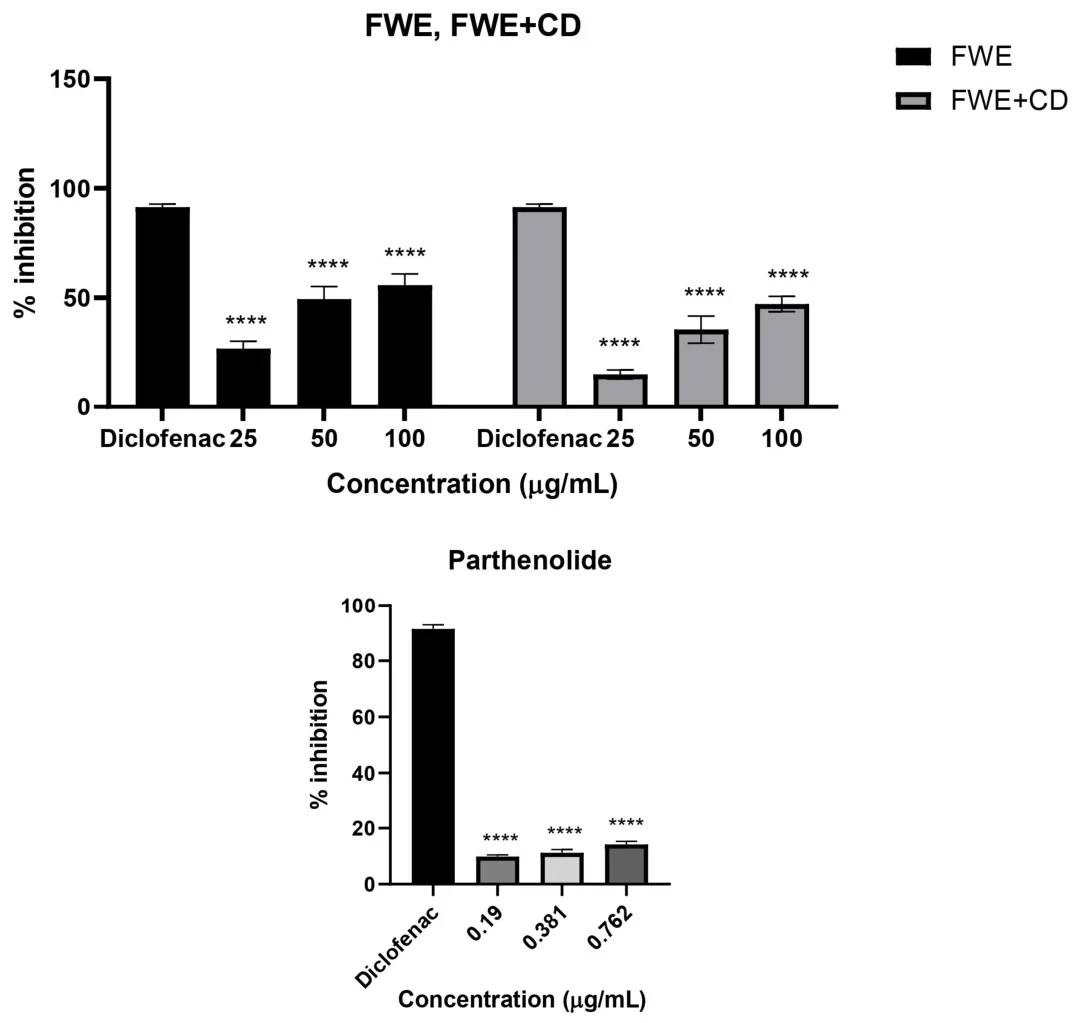
The impact of feverfew extract (FWE) and the extract in cyclodextrin (FWE+CD) at a range of concentrations (25, 50, and 100 µg/mL) and pure parthenolide (0.19–0.762 µg/mL) on erythrocyte membrane stabilization (heat-induced lysis of erythrocytes). One-way analysis of variance (ANOVA) with Tukey’s multiple comparison post hoc test (n = 3, **** *p* < 0.0001 vs. control); positive control, diclofenac 75 µg/mL.

The anti-inflammatory (membrane stabilization) potential of FWE and FWE+CD, as well as pure CD and PAR, was evaluated using the erythrocyte membrane stabilization assay. This mechanism is considered indicative of lysosomal membrane stabilization and subsequent suppression of inflammatory processes. As presented in Figure 8, both FWE and FWE+CD samples exhibited a moderate, but consistent, concentration-dependent anti-inflammatory effect. At the highest tested concentration (100 μg/mL), erythrocyte lysis was inhibited by approximately 47.2–55.9%, while lower concentrations showed weaker but measurable effects, confirming their ability to stabilize biological membranes. Although the activity is lower than that of diclofenac, which serves as a reference anti-inflammatory drug, the observed effect remains relevant for plant-derived formulations. The ability of the tested samples to stabilize erythrocyte membranes suggests their potential to protect lysosomal membranes, thereby limiting the release of pro-inflammatory mediators and contributing to anti-inflammatory effects. These results are consistent with the literature on *T. parthenium*, in which anti-inflammatory effects are primarily attributed to PAR via the NF-κB signaling pathway and pro-inflammatory mediators [65]. This activity is consistent with previously reported data on feverfew extracts, where anti-inflammatory effects are attributed to the combined action of PAR and polyphenolic compounds [4,55]. Moreover, pure PAR showed lower inhibition than FWE and FWE+CD at the tested concentrations, while CD alone did not affect erythrocyte membrane stability; therefore, the data are not shown graphically. These results suggest that the anti-inflammatory activity of FWE is not solely related to PAR, but likely results from the combination of PAR, polyphenols, and other bioactive constituents [4,55]. PAR is known to inhibit NF-κB activation and reduce the expression of pro-inflammatory cytokines, such as TNF-α and IL-6 [4,65], while polyphenols contribute through antioxidant and membrane-stabilizing effects [66,67]. Importantly, encapsulation did not reduce the anti-inflammatory activity of FWE. This indicates that CDs preserve or even enhance the functional properties of bioactive compounds by improving their stability and dispersion [61].

The observed activity confirmed the multifunctional potential of feverfew-based formulations, arising from the combined effect of PAR, polyphenols, and other bioactive compounds [1,4]. Although FWE+CD showed slightly lower activity than FWE, CD complexation largely preserved the antioxidant and anti-inflammatory potential of the extract, while improving formulation solubility and stability, and reducing cytotoxicity [61,62,68].

This study confirms that CD-assisted extraction and spray-drying encapsulation are effective strategies for developing stable, biologically active feverfew-based formulations for pharmaceutical, and particularly dermal and cosmeceutical, applications. Encapsulation with CD improved formulation stability and slightly reduced cytotoxicity without compromising biological activity, highlighting its suitability as a carrier system. Namely, cyclodextrins can form host–guest inclusion complexes, in which hydrophobic or partially hydrophobic regions of parthenolide and other phytochemicals are incorporated into the cyclodextrin cavity. This molecular association reduces their direct exposure to oxygen, moisture, light, and elevated temperature, thereby limiting oxidation, hydrolysis, and photo- or thermally induced degradation. In addition, encapsulation may reduce the volatility and chemical reactivity of sensitive constituents and promote their more uniform dispersion within the cyclodextrin matrix. The resulting protection and gradual release of bioactive compounds can preserve their antioxidant capacity and support sustained anti-inflammatory activity. Therefore, the observed biological performance of the developed particles is likely related not only to the initial concentration of feverfew-derived compounds but also to the stabilizing effect of cyclodextrin inclusion complexes during the product’s preparation, storage, and release.

As mentioned above, parthenolide has been reported to modulate several inflammation-related signaling pathways, including NF-κB and MAPK signaling, and to reduce the expression of pro-inflammatory mediators such as COX-2 and selected cytokines. Its activity may also be associated with the regulation of oxidative stress through pathways involving Nrf2. However, these mechanisms were not directly evaluated in the present study; therefore, the potential anti-inflammatory effects of the developed formulations should be interpreted as a hypothesis based on the reported biological activity of parthenolide and other feverfew constituents. Future in vitro and in vivo studies should investigate these molecular pathways and confirm the anti-inflammatory efficacy of the developed formulations.

The potential dermo-cosmetic applicability of the developed formulations is supported by the observed cytocompatibility toward keratinocytes under the tested conditions. Nevertheless, cytotoxicity represents only an initial safety assessment and does not establish dermal suitability or clinical safety. Skin permeation, topical stability, compatibility with cosmetic formulations, irritation potential, sensitization, and long-term dermatological safety were not investigated in the present study. Therefore, the dermo-cosmetic potential of these materials should be considered preliminary, and further studies using ex vivo or in vitro skin permeation models, stability protocols, formulation-compatibility tests, and extended dermatological safety assessments are needed before their practical use in topical products.

## 3. Materials and Methods

### 3.1. Chemicals

The 2-hydroxypropyl-β-cyclodextrin (CD) (97% purity, analytical grade) was provided by Acros Organics (Geel, Belgium). All reagents used for analytical procedures were of analytical grade: Folin–Ciocalteu reagent, gallic acid, catechin, sodium carbonate, sodium nitrite, sodium hydroxide, and aluminum chloride were purchased from Sigma-Aldrich Chemie GmbH (Munich, Germany). Absolute ethanol (99.8% purity), used for extraction procedures, was obtained from Fisher Scientific (Waltham, MA, USA). Ultrapure water was prepared using a Milli-Q purification system (Bedford, MA, USA), and HPLC-grade acetonitrile was obtained from Merck (Darmstadt, Germany). PAR standard was supplied by Millipore (Bedford, MA, USA). Thiazolyl blue tetrazolium bromide (MTT reagent, 1 mg/mL), sodium dodecyl sulfate (SDS), dimethyl sulfoxide (DMSO), methanol, and phosphate-buffered saline (PBS), which were used in the cell-based assays, were purchased from Sigma-Aldrich (St. Louis, MO, USA).

### 3.2. Plant Material

The dried flowers of feverfew (*T. parthenium*) were obtained in 2024 from the Production Sector of the Institute for Medicinal Plants Research from “Dr. Josif Pančić’’ (Belgrade, Serbia). Before further processing, the dried flowers were ground using a laboratory mill (IKA M 20 Universal Mill, IKA-Werke GmbH & Co. KG, Staufen, Germany) to obtain a homogeneous plant powder prior to extraction. Obtained plant powders were sieved through 63, 150, and 500 µm mesh sieves, following the specifications of the Yugoslavian Pharmacopoeia [69], to obtain a moderately fine powder with a particle size of 63–150 µm. The prepared samples were stored in airtight, light-protected glass containers under controlled room temperature conditions until further experiments. Before the extraction process, the dry matter content of the prepared sample was determined using a Kern & SOHN Moisture Analyzer GmbH (Balingen, Germany).

### 3.3. Maceration Extraction (ME) vs. Ultrasound-Assisted Extraction (UE)

To obtain an extract with optimal characteristics and a high bioactive compound content, the extraction procedures, maceration (ME) and ultrasound-assisted extraction (UE), were optimized and screened through comparative evaluation. ME was performed using an orbital shaker (Heidolph Promax 2020 shaker, Heidolph Instruments, Schwabach, Germany), at 180 rpm, at 25 °C, for 2 h. UE was performed in an ultrasonic bath (SONIC 4GT, VIMS Elektrik, Tršić, Serbia) for 1 h, with a constant frequency (35 kHz) and power (320 W). The ultrasonic bath was operated using the manufacturer’s fixed power settings; therefore, ultrasound power was not considered as an independent optimization parameter in the experimental design. During UE, the temperature was continuously monitored and maintained at 25 °C to minimize the thermal degradation of heat-sensitive bio-compounds, particularly PAR, by periodic addition of water to the ultrasonic bath. The temperature fluctuations during the extraction process were minimal and remained within the range of 24 to 26 °C. Furthermore, any increase in temperature was corrected by distilled water in addition to the ultrasonic bath. For each extraction technique, three green solvent systems were tested: water, 50% ethanol, and 70% ethanol (*v*/*v*). The solid–solvent ratio was set at 1:30, while the total volume of the extraction mixture was 25 mL. The solid–solvent ratio, ethanol concentration, and extraction time were selected based on preliminary screening results and according to the available literature [27]. In the next step, the extraction procedure was performed, with 0.5% (*w*/*w*) CD as a potential extraction “booster” (co-solvent) for each solvent combination. Overall, 6 samples were prepared for each extraction technique, with and without CD (Scheme 1).

The influence of the investigated extraction parameters was statistically evaluated using analysis of variance (ANOVA), and significant differences among extraction conditions were determined. The selection of the most promising extract was based on the combined evaluation of total polyphenol content, total flavonoid content, and PAR concentration determined by HPLC. All extraction experiments were performed in triplicate to ensure analytical reliability.

#### 3.3.1. Determination of Total Polyphenol Content

Total polyphenol content (TPC) was determined using a modified Folin–Ciocalteu procedure [70]. Briefly, 200 μL of extract was mixed with 1.0 mL of 10% Folin–Ciocalteu reagent, followed by 800 μL of 7.5% Na_2_CO_3_ after 4 min. After 2 h of incubation in the dark, absorbance was recorded at 765 nm using a UV–Vis spectrophotometer (Shimadzu UV-2600, Kyoto, Japan). A mixture of all other components, except for the sample (extract), was used as the blank. TPC was calculated from a gallic acid calibration curve (A = 0.10281 × C, r > 0.99), with concentrations ranging from 0 to 100 mg/L, and expressed as mg gallic acid equivalents per gram of dried plant mass (mg GAE/g).

#### 3.3.2. Determination of Total Flavonoid Content

Total flavonoid content (TFC) was quantified spectrophotometrically using a modified aluminum chloride colorimetric assay [71]. Briefly, 200 μL of extract was diluted with 800 μL of distilled water, followed by the addition of 60 μL of 5% (*w*/*v*) sodium nitrite, and after 5 min, 60 μL of 10% (*w*/*v*) AlCl_3_. Subsequently, 400 μL of 1 M NaOH was added after 6 min, and the final volume was adjusted to 2 mL with distilled water. For blank correction, a control mixture containing all reagents except the extract was prepared. The absorbance was measured at 510 nm. TFC was expressed as mg catechin equivalents per gram of dried plant mass (mg CE/g).

#### 3.3.3. Determination of PAR by HPLC

PAR content was determined by HPLC (Agilent 1100 Series, Santa Clara, CA, USA) with a reversed-phase C18 column Zorbax XDB-C1815. Separation was performed under isocratic conditions with a mobile phase of acetonitrile and ultrapure water (45:55, *v*/*v*) at a flow rate of 1.0 mL/min. The injection volume was 10 μL, while the column and autosampler were maintained at 25 °C. Detection was performed at 210 nm. All samples were analyzed at least in duplicate to verify analytical reproducibility. Quantification was performed by external standard calibration with PAR reference solutions, and the results are expressed as mg PAR per g of dried plant mass (mg PAR/g).

### 3.4. Extract Preparation

After extraction optimization, the selected extract with the highest polyphenol, flavonoid, and PAR contents was prepared in a larger volume for encapsulation. Extraction was performed using an eco-friendly UE protocol for 1 h at a solid–solvent ratio of 1:30 with 50% (*v*/*v*) ethanol, containing 0.5% CD. After the extraction process, ethanol was removed using a rotary evaporator (IKA, HB4-basic, Staufen im Breisgau, Germany) under vacuum at 50 °C to prevent the degradation of sensitive compounds, particularly PAR. The obtained liquid feverfew extract (LFWE), with a dry residue of 4.2% (Kern & SOHN Moisture Analyzer GmbH, Balingen, Germany) and an alcohol content of 3.5% (*w*/*w*) (determined according to the European Pharmacopoeia method (Ph. Eur. 2.9.10) [56]) was used for the spray-drying method.

### 3.5. Encapsulation Process

The spray-drying technique was used to obtain FWE and FWE+CD powders. Spray drying was performed using a Labtex ESDTi spray dryer (Labtex, Huddersfield, UK), with a 0.5 mm nozzle diameter, under the following conditions: air flow rate = 70 m^3^/h, liquid feed flow rate = 11 mL/min, atomization pressure = 2.5 bar, inlet temperature = 135–136 °C, and outlet temperature = 56–60 °C. The spray-drying conditions were optimized during the process. One portion of the LFWE was spray-dried without a carrier, leading to pure FWE powder, while another portion was spray-dried with a carrier, yielding a CD-containing feverfew extract (FWE+CD). The CD, 40% (*w*/*w*), was dissolved in LFWE and stirred for 24 h before encapsulation. The two feed formulations were spray-dried under identical conditions (Scheme 1). The obtained spray-dried powders were kept in high-density glass bottles in a desiccator at room temperature until further analyses.

### 3.6. Analysis of Powder Properties

The obtained powders were comprehensively evaluated with respect to their technological, physical, structural, morphological, and chemical properties. Technological characterization included the determination of process yield and residual moisture content of powders. Advanced physicochemical characterization was performed using several analytical techniques: structural features and potential intermolecular interactions were examined by Fourier-transform infrared (FTIR) spectroscopy; crystalline and amorphous domains were assessed by X-ray diffraction (XRD) analysis; energy-dispersive X-ray spectroscopy (EDS), coupled with scanning electron microscopy (SEM), was used to analyze the elemental composition of the sample and particle morphology; particle size distribution was determined by laser diffraction particle size analysis; and thermal stability was assessed by differential scanning calorimetry (DSC) analysis. The TPC and TFC were quantified spectrophotometrically by UV–VIS analysis, whereas PAR concentration was determined using HPLC. In addition, the biological performance of the spray-dried powders was assessed using antioxidant, anti-inflammatory, and cytotoxicity assays to establish the functional and biopharmaceutical relevance of the developed delivery system with FWE.

#### 3.6.1. Technological Powder Characterization

##### Powder Yield

The production yield (Y) of the spray-drying/encapsulation process was determined by dividing the actual mass of the collected dried extract ($m_{extract}$) by the theoretical mass expected after the drying process, based on total solids present in the initial liquid feed ($m_{theor}$):

(1) $$Y\;(\%)\;=\frac{\;M_{extract}\;}{M_{theor}}\;\times \;100$$

The yield of FWE is given as follows:

(2) $$Y_{FWE}\;(\%)\;=\frac{\;M_{FWE}\;}{X_{LFWE}{\;\times \;M}_{LFWE}}\;\times \;100$$

where $M_{FWE}$ is the actual mass of FWE, $M_{LMFE}$ is the total mass of the liquid feed extract sprayed and $X_{LFWE}$ is the mass fraction of solid materials in the initial feed (LFWE).

The yield of FWE+CD is given as follows:

(3) $$Y_{FWE+CD}\;(\%)\;=\frac{\;M_{FWE+CD}\;}{{M_{CD}\;+\;X}_{LFWE}{\;\times \;M}_{LFWE}}\;\times \;100$$

where $M_{FWE+CD}$ is the actual mass of FWE+CD and $M_{CD}$ is the mass of carrier (CD) added to the feed liquid extract.

##### Moisture Content

The residual moisture content of the prepared powders was determined by a gravimetric method using a halogen moisture analyzer (Kern & SOHN Moisture Analyzer GmbH, Balingen, Germany). Samples were heated at 105 °C under controlled conditions until a constant mass was achieved. The results were expressed as percentage weight by weight (% *w*/*w*).

##### Bulk and Tapped Densities

Bulk density of the spray-dried powders was determined following a previously reported procedure [11]. Briefly, 1 g of the sample was placed into a 5 mL graduated glass cylinder. The cylinder was subjected to controlled agitation at 300 rpm for 5 min using a Unimax 1010 shaker (Heidolph Instruments GmbH & Co. KG, Schwabach, Germany) at room temperature to ensure uniform settling of the powder bed. The bulk density was calculated from the occupied volume and expressed in mg/mL. Tapped density was determined after 120 mechanical taps, followed by re-measurement of the powder volume. Powders’ flowability, compressibility, and cohesiveness properties were evaluated using Carr’s index (CI) and the Hausner ratio (HR), calculated from bulk and tapped density values according to standard equations [72]:

(4) CI = (ƍ tapped − ƍ bulk) / ƍ tapped × 100

(5) HR=ƍ tapped / ƍ bulk

where ƍ_tapped and ƍ_bulk represent the tapped and bulk densities, respectively.

##### Rehydration Time and pH Measurements

Rehydration time was determined as the time required for complete dissolution of the spray-dried powder in water at room temperature. Briefly, 250 mg of powder was dispersed in 5 mL of distilled water under continuous magnetic stirring, and the time required to achieve full reconstitution, defined as the absence of visible undissolved particles, was expressed in seconds (s) [10]. The pH value of each sample (50 mg/mL) was measured using a calibrated pH meter (HI 99161, Hanna Instruments, Woonsocket, RI, USA).

#### 3.6.2. Physicochemical Characterization of Spray-Dried Powders

##### Powder Composition Analysis by FTIR Spectroscopy

Fourier-transform infrared (FTIR) spectroscopy was employed to evaluate structural changes and interactions in FWE, FWE+CD, and the pure carrier material. Spectra were recorded using a Cary 630 FTIR spectrometer (Agilent, Santa Clara, CA, USA) in the range of 400–4000 cm^−1^ at room temperature. The obtained spectra were analyzed to identify characteristic functional group vibrations and spectral shifts indicative of inclusion complex formation.

##### Powders Thermal Characterization by Differential Scanning Calorimetry

Thermal properties of the samples were evaluated using a DSC Q10 by TA Instruments (New Castle, DE, USA). Samples were placed in aluminum pans and hermetically sealed, with an empty pan used as a reference, and heated from 20 °C to 300 °C using a linear temperature ramp at 10 °C/min. Endothermic and exothermic transitions were recorded, and thermal transition temperatures and enthalpy changes (J/g) were determined after baseline correction. Data were analyzed using Universal Analysis software (v9.9 Build 303, TA Instruments, New Castle, DE, USA).

##### Particle Size Distribution

Particle size distributions of the spray-dried powders were determined by laser diffraction using a Mastersizer 2000 (Malvern Instruments, Worcestershire, UK). The results were expressed as d(0.1), d(0.5), and d(0.9), representing the diameters below which 10%, 50%, and 90% of the total particles are found, respectively. The distribution width was evaluated using the SPAN value:

(6) $$SPAN=\frac{d_{0.9}-d_{0.1}}{d_{0.5}}$$

where $d_{0.1}$, $d_{0.5}$, and $d_{0.9}$ are the threshold diameters where 10%, 50%, and 90% of the total sample volume is made up of smaller particles. The surface-weighted mean diameter (D [3,2]) and the volume-weighted mean diameter (D [4,3]) were additionally determined for particle size and dispersion behavior.

##### X-Ray Diffraction (XRD) Analysis of Powders 

XRD analysis was performed to characterize the crystallographic properties of the samples’ wide-angle XRD patterns, recorded using a Bruker D2 Phaser X-ray diffractometer (Bruker AXS, Karlsruhe, Germany), equipped with a 1-dimensional Lynxeye detector, and Ni-filtered Cu Kα radiation run at 30 kV and 10 mA. Patterns were collected from 5 to 55° 2θ using a step size of 0.02.

##### Scanning Electron Microscopy (SEM) and Energy-Dispersive X-Ray Spectroscopy (EDS) Analysis

The surface morphology was analyzed using the SEM device JSM-7800F (JEOL Ltd., Tokyo, Japan). For X-ray microanalysis of powders (energy-dispersive spectroscopy), an Ultim Max EDS detector (Oxford Instruments, High Wycombe, Buckinghamshire, UK) was used. Imaging was performed at an acceleration voltage of 5 kV with a beam current of 200 pA, while EDS measurements were carried out at 15 kV and a beam current of 800 pA.

#### 3.6.3. Chemical Characterization of Spray-Dried Powders

Approximately 0.05 mg of all obtained powders was dissolved in 10 mL of distilled water using a Vortex mixer (Vortex 3, IKA, Staufen, Germany) for chemical analysis. The resulting samples were further analyzed. Samples were analyzed for TPC, TFC, and PAR content according to the procedures described above. Analysis of TPC, TFC, and PAR was performed using UV–VIS and HPLC techniques, respectively. TPC, TFC, and PAR were expressed as mg GAE/g, mg CE/g, and mg PAR/g of the spray-dried powders.

#### 3.6.4. Biological Activity Examination of Spray-Dried Powders

##### Cell Culture

The HaCaT cell line, consisting of spontaneously immortalized human keratinocytes, was generously supplied by the Institute for Biological Research “Siniša Stanković”, National Institute of the Republic of Serbia, University of Belgrade, Belgrade, Serbia. HaCaT human keratinocytes were kept in 25 cm^2^ tissue culture flasks in a humidified incubator at 37 °C with 5% CO_2_. They were grown in a complete medium containing Dulbecco’s Modified Eagle’s Medium/Nutrient Mixture F-12 Ham (DMEM F12, Biowest, Nuaillé, France), 10% fetal calf serum (FCS, Gibco, Waltham, MA, USA), and 1% antibiotic–antimycotic solution (Capricorn Scientific GmbH, Ebsdorfergrund, Germany). After reaching 70% confluence, the cells were trypsinized (0.25% trypsin-EDTA solution, Institute for Virology, Vaccines, and Serum “Torlak”, Belgrade, Serbia), seeded in 96-well plates (1.5 × 10^4^ cells/well) and allowed to attach to the wells for 24 h at 37 °C, with 5% CO_2_, before the treatment.

##### Cytotoxicity Evaluation of Spray-Dried Powders

The influence of FWE, FWE+CD, PAR standard, and the carrier (CD) on the keratinocyte viability was investigated [73]. The HaCaT cells in complete RPMI medium were seeded in 96-well plates at a density of 1.5 × 10^4^ cells/well, in a final volume of 100 µL per well. The medium was exchanged after 24 h, and treatments were added in a total volume of 100 µL/well. Following incubation with the treatments or solvent (DMSO vehicle control containing a matched DMSO concentration) at 37 °C for 24 h, an MTT assay was performed. MTT reagent (10 µL per well) was added, and the cells were incubated in the dark at 37 °C for 2 h for the reaction to occur. Further, purple formazan crystals were dissolved with SDS. Finally, the absorbance was measured at 570 nm on a microplate reader (Epoch, BioTek, Winooski, VT, USA) after the complete solubilization of the crystals. Data were expressed as percentage viability compared to the control (100%), as mean values represented on bars.

##### Antioxidant Analysis of Spray-Dried Powders Using the H_2_DCFDA Assay

HaCaT cells were seeded and allowed to attach overnight in a humidified incubator at 37 °C with 5% CO_2_. On the following day, the culture medium was replaced, and the cells were treated with samples (FWE, FWE+CD, CD, and PAR standard) at final concentrations of 12.5, 25, 50, 100, and 200 µg/mL in complete medium (100 µL per well). After 24 h of incubation, the treatments were removed, and the cells were washed with PBS. The intracellular reactive oxygen species (ROS) levels were assessed according to the manufacturer’s instructions [74]. Briefly, the cells were incubated with 5 µM of the cell-permeable, oxidation-sensitive probe H_2_DCFDA (Merck Millipore, Darmstadt, Germany) diluted in PBS for 45 min in the dark. Following incubation, the cells were washed with PBS and then exposed to PBS alone (negative control) or 200 µM H_2_O_2_ (positive control). After 2 h of incubation, the oxidation of non-fluorescent H_2_DCFDA to the highly fluorescent 2′,7′-dichlorofluorescein (DCF) was measured using a fluorescence plate reader (Wallac 1420 Multilabel Counter Victor 3V, PerkinElmer Life and Analytical Sciences, Boston, MA, USA) at excitation and emission wavelengths of 485 nm and 535 nm, respectively. Fluorescence intensity was expressed as relative fluorescence units (RFUs). Data are presented as mean values.

##### Anti-Inflammatory (Membrane Stabilization) Examinations of Spray-Dried Powders

To assess the anti-inflammatory potential through membrane stabilization under in vitro conditions, the red blood cell (RBC) membrane stabilization assay was utilized [75]. Erythrocyte membranes resemble lysosomal membranes, making them suitable for evaluating the effects of the extract and its encapsulated formulations on membrane integrity. Blood samples were collected from the Institute for the Application of Nuclear Energy (INEP), Belgrade, Serbia (biochemical laboratory). The study protocol received approval (0203-07-013/005/2025, 14 February 2025) from the ethical committee of INEP, University of Belgrade, and informed consent was obtained from all donors. Sodium oxalate was used to prevent coagulation. The collected blood was refrigerated for 24 h and then centrifuged at 2500 rpm for 5 min using a Thermo Scientific Sorvall WX Ultra series centrifuge (Thermo Fisher Scientific, Waltham, MA, USA). The erythrocytes were washed with sterile 0.9% NaCl solution, followed by repeated centrifugation. This washing procedure was performed three times, and the packed cell volume was measured; the cells were resuspended in PBS (10 mM, pH 7.4) to obtain a 40% (*v*/*v*) erythrocyte suspension. Hemoglobin content in the suspension was quantified spectrophotometrically at 560 nm using a UV-1800 spectrophotometer (Shimadzu, Kyoto, Japan). Diclofenac (75 μg/mL, Galenika, Belgrade, Serbia) served as the positive control. Anti-inflammatory activity was expressed as the percentage inhibition of RBC lysis. Aliquots of isotonic buffers containing 25, 50, or 100 μg/mL of the test samples (5 mL each) were placed in centrifuge tubes. An equal volume of buffer without a sample was used as the control. Erythrocyte suspension (50 μL) was added to each tube, followed by gentle mixing. Two tubes were incubated in a water bath at 54 °C for 20 min, while the remaining two tubes were maintained in an ice bath (0–5 °C) for the same duration. After incubation, samples were centrifuged at 5000 rpm for 5 min, and the absorbance of the supernatant was measured at 560 nm. The percentage inhibition of RBC lysis was calculated using the following formula:

(7) $$\%\;inhibition\;of\;RBC\;lysis\;=\;100\;\times \;(1\;-\;\frac{{OD}_{2}-{OD}_{1}}{{OD}_{3}-{OD}_{1}})$$

OD_1_—non-heated sample; OD_2_—heated sample; OD_3_—heated control.

### 3.7. Statistical Analysis

Extraction procedures and all chemical analyses were performed in triplicate, as were the analyses of technological and physicochemical characterization. Numerical data are expressed as means ± standard deviation. Biological performance of the formulations was evaluated in three independent experiments performed in triplicate (n = 9), with mean values presented in bar graphs. Statistical significance was assessed using one-way analysis of variance (ANOVA), followed by Tukey’s post hoc test for multiple comparisons.

## 4. Conclusions

This study has successfully demonstrated the feasibility of integrating solvent extraction and spray-drying-based microencapsulation into a highly effective technology for stabilizing bioactive compounds derived from feverfew. The use of CD as both a co-extractant and an encapsulation carrier proved effective in enhancing the recovery, stability, and functional performance of polyphenols and PAR-rich extracts. The ultrasound-assisted extraction, using 50% (*v*/*v*) ethanol and 0.5% CD, was identified as the most efficient for maximizing bioactive yield under environmentally favorable conditions. The subsequent spray-drying encapsulation step produced powders with improved technological characteristics, including higher process yield, reduced residual moisture, and suitable flow behavior, thereby confirming the robustness of the developed technology. Notably, the CD-based microcapsules exhibited enhanced physicochemical stability and biological activities, supporting their potential application as stable delivery systems for sensitive phytochemicals. FTIR and DSC results support the successful formation of a CD-based encapsulated system and suggest preservation and stabilization of PAR within the spray-dried powder. This structural organization likely contributes to enhanced protection of thermolabile constituents, including parthenolide (PAR), during processing. The results highlight the multifunctional role of cyclodextrins in plant extract formulation and support their potential application in the development of advanced phytopharmaceutical and nutraceutical delivery systems. Compared with other micro- and nano-encapsulated systems (liposomes, polymeric nanoparticles, nanoemulsions, solid lipid nanoparticles and nanostructured lipid carriers), HPβCD-based spray drying offers a simpler, scalable, and industrially applicable approach for the stabilization of plant extracts. Although these mentioned encapsulation systems can improve the bioavailability of bioactives, they generally require more complex formulations, specialized excipients, and multistep manufacturing processes. In contrast, the HPβCD-based extraction and spray-drying technique simultaneously enhances the aqueous solubility of poorly soluble phytochemicals through inclusion complex formation and protects bioactive compounds from thermal and oxidative degradation during spray drying. Therefore, the integrated combination of UE and HPβCD-assisted spray drying developed in this study provides a green, cost-effective, and pharmaceutically relevant strategy for the stabilization and delivery of parthenolide- and polyphenol-rich feverfew extracts. Both the non-encapsulated extract (FWE) and the cyclodextrin-based formulation (FWE+HPβCD) retained significant biological activity, while encapsulation improved physicochemical stability and formulation performance. The combined antioxidant and anti-inflammatory activities further underline the multifunctional therapeutic potential of the developed systems. Nevertheless, some limitations should be acknowledged. Although the physicochemical characterization confirmed the successful formation of the parthenolide–HPβCD inclusion system, further studies involving release kinetics, long-term stability evaluation, and in vivo studies could fully establish its therapeutic applicability. A comprehensive investigation of storage stability under different environmental conditions, and long-term changes in powder properties, will be the focus of a subsequent study based on the ongoing stability evaluation.

## Figures and Tables

**Table 1:** Parthenolide yield (Y_PAR_), total phenolic content (TPC), and total flavonoid content (TFC) of *Tanacetum parthenium* L. flower extracts obtained by maceration (ME) and ultrasound-assisted extraction (UE), in the presence and absence of hydroxypropyl-β-cyclodextrin (CD).

Type of Extraction and Extractant	Y_PAR_, mg PAR/g	TPC, mg GAE/g	TFC, mg CE/g
Maceration (ME)			
H_2_O	3.21 ± 0.26 ^c^	18.76 ± 2.75 ^d^	3.17 ± 0.40 ^c^
50% EtOH	5.65 ± 0.73 ^a^	73.11 ± 6.79 ^ab^	25.76 ± 3.47 ^a^
70% EtOH	5.70 ± 0.56 ^a^	62.21 ± 8.44 ^abc^	25.47 ± 2.37 ^a^
H_2_O + CD	3.56 ± 0.52 ^bc^	33.99 ± 3.42 ^d^	4.76 ± 0.50 ^bc^
50% EtOH + CD	5.81 ± 0.42 ^a^	64.63 ± 4.40 ^abc^	22.99 ± 2.57 ^a^
70% EtOH + CD	4.98 ± 0.55 ^ab^	64.64 ± 4.27 ^abc^	23.51 ± 2.15 ^a^
Ultrasound (UE)			
H_2_O	3.03 ± 0.41 ^c^	23.70 ± 3.12 ^d^	10.02 ± 1.47 ^b^
50% EtOH	5.78 ± 0.40 ^a^	60.75 ± 4.29 ^abc^	26.78 ± 2.40 ^a^
70% EtOH	5.76 ± 0.52 ^a^	51.79 ± 7.19 ^c^	24.21 ± 2.20 ^a^
H_2_O + CD	3.15 ± 0.44 ^c^	26.18 ± 2.42 ^d^	9.49 ± 0.55 ^b^
50% EtOH CD	6.30 ± 0.38 ^a^	75.76 ± 7.91 ^a^	28.59 ± 2.76 ^a^
70% EtOH CD	5.42 ± 0.49 ^a^	57.52 ± 7.73 ^bc^	25.29 ± 1.27 ^a^

Abbreviations: Parthenolide, PAR; gallic acid equivalent, GAE; catechin equivalent, CE. Different letters indicate statistically significant differences within the same column, according to Tukey’s test (*p* < 0.05).

**Table 2:** Technological properties of spray-dried FWE and FWE+CD.

Samples	Yield (%)	Moisture (%)	BD	TD	CI (%)	HR	pH	RT (s)
FWE	47.27 ± 2.62 ^b^	8.30 ± 1.16 ^a^	0.46 ± 0.05 ^a^	0.49 ± 0.06 ^a^	6.67 ± 0.45 ^b^	1.07 ± 0.09 ^a^	5.08 ± 0.48 ^a^	7.66 ± 0.79 ^b^
FWE+CD	65.94 ± 7.75 ^a^	6.94 ± 1.03 ^a^	0.23 ± 0.03 ^b^	0.26 ± 0.02 ^b^	13.48 ± 0.76^a^	1.16 ± 0.09 ^a^	5.16 ± 0.32 ^a^	52.40 ± 7.28 ^a^

Different letters indicate statistically significant differences within the same column according to Tukey’s test (*p* < 0.05). BD—bulk density, TD—tapped density, CI—Carr index, HR—Hausner ratio, RT—rehydration time.

**Table 3:** The transition temperatures and enthalpy changes in spray-dried feverfew extract (FWE), FWE encapsulated within 2-hydroxypropyl-β-cyclodextrin (FWE+CD), and the carrier 2-hydroxypropyl-β-cyclodextrin (CD).

Samples	T1	T2	∆H1	∆H2
FWE	90.35 ± 9.52 ^b^	160.43 ± 14.54 ^b^	224.70 ± 11.73 ^b^	68.99 ± 4.73 ^a^
FEW+CD	98.20 ± 6.23 ^ab^	179.82 ± 14.34 ^b^	274.80 ± 34.78 ^b^	32.75 ± 2.46 ^b^
CD	119.35 ± 12.37 ^a^	266.63 ± 23.97 ^a^	359.30 ± 35.63 ^a^	0.51 ± 0.06 ^c^

Abbreviations: T—temperature transition; ∆H—enthalpy change, Different letters indicate statistically significant differences within the same row according to Tukey’s test (*p* < 0.05).

## Data Availability

The original contributions presented in this study are included in the article. Further inquiries can be directed to the corresponding author.

## Supplementary Materials

The following supporting information can be downloaded at: https://www.mdpi.com/article/10.3390/ph19081176/s1. Figure S1: EDS analysis of (a) 2-hydroxypropyl-β-cyclodextrin (CD), (b) feverfew extract (FWE), and (c) feverfew extract encapsulated with CD (FWE+CD).

## Abbreviations

The following abbreviations are used in this manuscript:

FWE	Feverfew extract
CD	Hydroxypropyl-β-cyclodextrin
PAR	Parthenolide
