# Supplementary material for: Cyclodextrin-Assisted Extraction and Encapsulation of Parthenolide- and Polyphenol-Rich Feverfew (Tanacetum parthenium L.) for Enhanced Bioactivity
Source: Pharmaceuticals (Basel). 2026 Jul 27;19(8):1176. doi: 10.3390/ph19081176 (PMC13516332; doi:10.3390/ph19081176)
Supplement: Supplementary file 1 [file pharmaceuticals-19-01176-s001.zip › pharmaceuticals-4427612-supplementary.pdf]

Article

Cyclodextrin-Assisted Extraction and Encapsulation of Parthenolide- and Polyphenol-Rich Feverfew (*Tanacetum parthenium* L.) for Enhanced Bioactivity

Nada Ćujić Nikolić [1,†], Dajana Lazarević [2,*,†], Aleksandra Jovanović [3], Jelena Jovanović [2], Katarina Šavikin [1], Nibina Nizar [4], Gorana Ilić [3], Goran Vladisavljević [4] and Tatjana Trtić-Petrović [2]

[1] Institute for Medicinal Plants Research ''Dr. Josif Pančić'', 11000 Belgrade, Serbia; ncujic@mocbilja.rs

[2] Vinča Institute of Nuclear Sciences, National Institute of the Republic of Serbia, University of Belgrade, 11000 Belgrade, Serbia; ttrtic@vinca.rs

[3] Institute for the Application of Nuclear Energy INEP, University of Belgrade, 11080 Belgrade, Serbia; ajovanovic@inep.co.rs (A.J.); gorana.ilic@inep.co.rs (G.I.)

[4] Chemical Engineering Department, Loughborough University, Loughborough LE113TU, Leicestershire, UK;g.vladisavljevic@lboro.ac.uk

* Correspondence: dajana.lazarevic@vin.bg.ac.rs

† These authors contributed equally to this work.

a)

b)

c)

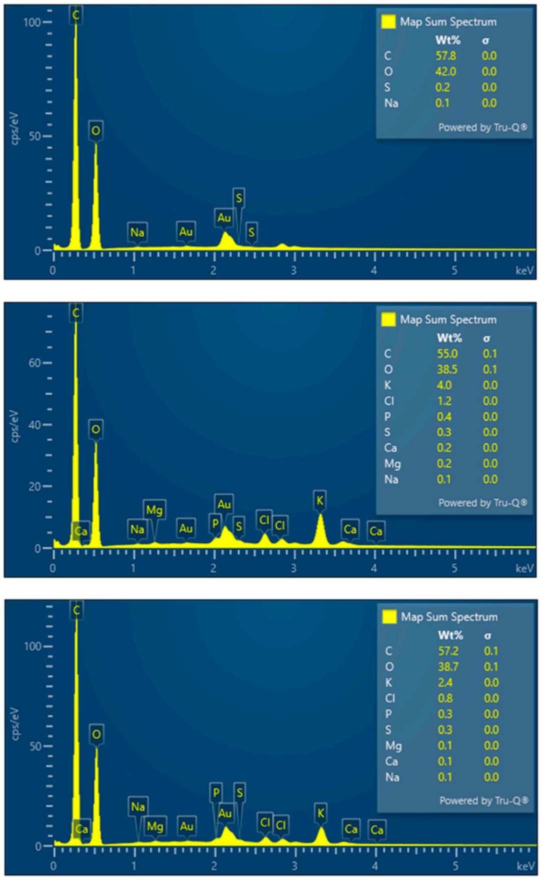

Figure S1. EDS analysis of a) 2-hydroxypropyl-β-cyclodextrin (CD), b) feverfew extract (FWE), and c) feverfew extract encapsulated with CD (FWE+CD).
